# Multiple myeloma-derived Jagged ligands increases autocrine and paracrine interleukin-6 expression in bone marrow niche

**DOI:** 10.18632/oncotarget.10820

**Published:** 2016-07-24

**Authors:** Michela Colombo, Serena Galletti, Gaetano Bulfamante, Monica Falleni, Delfina Tosi, Katia Todoerti, Elisa Lazzari, Leslie A. Crews, Catriona H.M. Jamieson, Sara Ravaioli, Francesco Baccianti, Silvia Garavelli, Natalia Platonova, Antonino Neri, Raffaella Chiaramonte

**Affiliations:** ^1^ Department of Health Sciences, Università degli Studi di Milano, Milano, Italy; ^2^ Department of Oncology and Hemato-Oncology, Università degli Studi di Milano, Hematology Unit, Fondazione Cà Granda IRCCS Policlinico, Milano, Italy; ^3^ Unit of Pathology A.O. San Paolo, Milan, Italy; ^4^ Laboratory of Pre-Clinical and Translational Research, IRCCS-CROB, Referral Cancer Center of Basilicata, Rionero in Vulture, Italy; ^5^ Department of Medicine, Division of Regenerative Medicine, Moores Cancer Center at University of California, San Diego, La Jolla, CA, USA; ^6^ Sanford Consortium for Regenerative Medicine, La Jolla, CA, USA

**Keywords:** multiple myeloma, Notch signaling, Jagged, interleukin-6, bone marrow niche, Pathology Section

## Abstract

Multiple myeloma cell growth relies on intrinsic aggressiveness, due to a high karyotypic instability, or on the support from bone marrow (BM) niche.

We and other groups have provided evidences that Notch signaling is related to tumor cell growth, pharmacological resistance, localization/recirculation in the BM and bone disease.

This study indicates that high gene expression levels of Notch signaling members (*JAG1, NOTCH2, HES5* and *HES6*) correlate with malignant progression or high-risk disease, and Notch signaling may participate in myeloma progression by increasing the BM levels of interleukin-6 (IL-6), a major player in myeloma cell growth and survival. Indeed, *in vitro* results, confirmed by correlation analysis on gene expression profiles of myeloma patients and immunohistochemical studies, demonstrated that Notch signaling controls IL-6 gene expression in those myeloma cells capable of IL-6 autonomous production as well as in surrounding BM stromal cells. In both cases Notch signaling activation may be triggered by myeloma cell-derived Jagged ligands. The evidence that Notch signaling positively controls IL-6 in the myeloma-associated BM makes this pathway a key mediator of tumor-directed reprogramming of the bone niche.

This work strengthens the rationale for a novel Notch-directed therapy in multiple myeloma based on the inhibition of Jagged ligands.

## INTRODUCTION

Multiple Myeloma (MM) is a clonal B-cell malignancy that affects post-germinal center plasma cells (PCs) and represents about 13% of all hematologic cancers [1]. Plasma cell dyscrasias such as MM are characterized by clonal proliferation of PCs in the bone marrow (BM). The interaction between MM cells and BM niche components plays a key role in MM [2, 3]. Indeed, MM cells and BM stromal cells (BMSCs) interact through adhesion molecules and soluble factors (such as cytokines and growth factors) [4]. The activation of BMSCs mediated by neoplastic cells further supports the survival and growth of tumor cells and promotes the osteolytic damage associated with MM [2, 3].

Interleukin-6 (IL-6) is one of the most important factors for MM cell growth and survival *in vivo* [4, 5]. There are two different sources of IL-6 in MM that promote tumor development and maintenance *in vivo*: autocrine IL-6 derived from myeloma cells [6–9] and paracrine IL-6 derived from non-malignant cells of the BM niche [10–12]. BMSCs produce high levels of IL-6 that directly stimulates survival and proliferation of malignant PCs [5]. The relevance of IL-6 in MM disease is supported by multiple lines of evidence. Specifically, high levels of IL-6 in the serum of MM patients: i) directly correlate with tumor burden and disease severity [13, 14]; ii) promote MM progression [15, 16] through pleiotropic effects on cell proliferation, survival, migration and resistance to conventional chemotherapy; and iii) may represent a negative prognostic factor [17–19]. Moreover, anti-IL-6 monoclonal antibody exposure may transiently reverse MM manifestations [20].

Several studies indicate that intracellular production of IL-6 is mainly regulated at a transcriptional level. In particular, the nuclear factor *k*B (NF-*k*B), activated by diverse stimuli such as interleukin-1 (IL-1) and tumor necrosis factor a (TNFα) [21–24], plays a key role in regulating IL-6 expression. Moreover, recent evidence has further highlighted the ability of the Notch pathway to control IL-6 expression in hematopoietic stem cells [25, 26] and in macrophages [27].

In mammals, the Notch pathway consists of four transmembrane receptors (NOTCH1-4) and two families of ligands, the Serrate-like ligands (JAGGED1 and 2) and the Delta-like-ligands (DLL-1/3/4) [28]. Ligand binding induces two proteolytic cleavage events that release the cytoplasmic portion of the receptors (the active intracellular Notch, ICN), allowing its translocation to the nucleus [29]. Nuclear ICN regulates transcription of several target genes involved in physiologic development and cancer, such as the transcriptional factors *Hairy and Enhancer of Split* (*HES1-7* and *HEY1-2, L*) [30]. Deregulation of the Notch pathway has been associated with the pathogenesis of several solid tumors (i.e. glioblastoma, melanoma and breast, colorectal and pancreatic cancer) [31] and hematologic malignancies (i.e. T-ALL, B-ALL, AML, B-CLL and MM) due to activating mutations or putative epigenetic mechanisms [28, 32–35].

Notably, immunohistochemical studies have shown that MM cells overexpress the receptors NOTCH1 and 2 and the two ligands JAGGED1 and JAGGED2 [36–39]. In particular, JAGGED1 is overexpressed during the progression from monoclonal gammopathy of uncertain significance (MGUS) to MM [38], whereas JAGGED2 dysregulation is an earlier event that precedes MGUS and is correlated with disease stage [40]. The molecular mechanisms underlying JAGGED2 dysregulation have been investigated and involve epigenetic alterations [36] or overexpression of the ubiquitin ligase Skeletrophin, acting in JAGGED2 processing [39].

Several lines of evidence from our work and other groups highlighted the importance of Notch signaling in MM pathogenesis [28, 41], thus providing a strong rationale for its use as a possible therapeutic target. Indeed, Notch inhibition induces tumor cell apoptosis, reduces drug resistance [42–44], self-renewal [45], and bone disease [46–48], and impairs the migration/recirculation of malignant PCs to the BM [49].

This work builds upon experimental evidence showing that Notch pathway dysregulation in myeloma cells mediates key interactions between MM cells and BMSCs. Additionally, upregulation of Notch signaling members correlates with MM progression or high-risk disease. Here, we provide new evidences that upregulated Notch signaling supports MM cell growth and contributes to disease progression by promoting activation of an IL-6 autocrine loop in MM and favoring IL-6 paracrine release by the surrounding BMSCs.

## RESULTS

### Notch signaling is deregulated during MM cell progression and in high-risk disease

**Figure 1:**
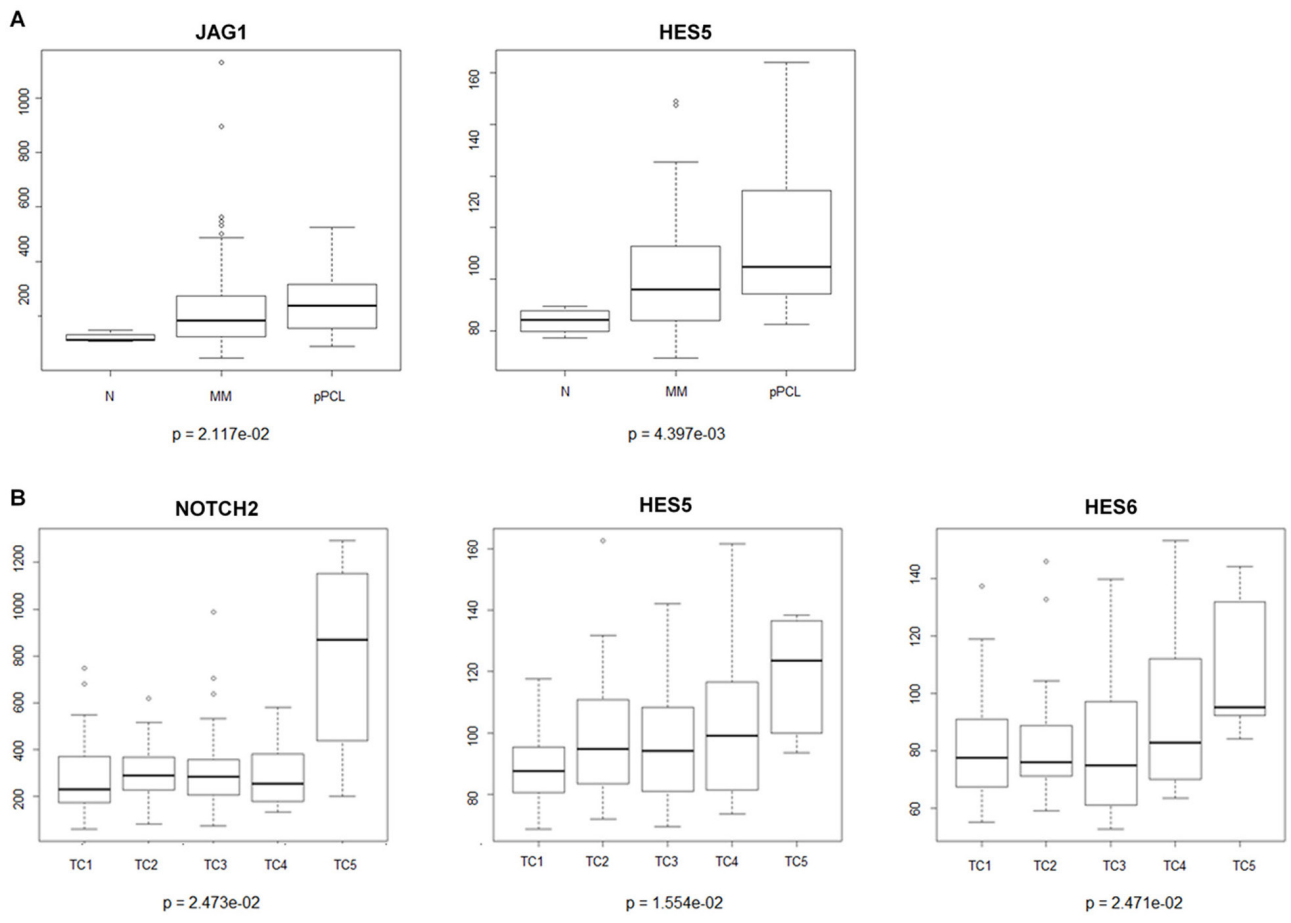
NOTCH related genes are overexpressed during MM progression Microarray expression levels of genes belonging to the NOTCH pathway in purified plasma cells (PCs) from normal controls (N), multiple myeloma (MM), and primary plasma cell leukemia (pPCL) samples. The absolute expression levels (linear scale) of *NOTCH2*, *JAG1*, *HES5* and *HES6* transcripts were assessed by means of microarray analysis. Box plot representations of messenger RNA expression levels in **A.** PC samples from 4 healthy donors (N), 129 MM and 24 pPCL patients; and **B.** 129 MM samples stratified according to TC classification (33 TC1, 28 TC2, 38 TC3,19 TC4 and 6 TC5). Kruskal-Wallis test was applied to assess statistical significance of differential gene expression profiles between all PC dyscrasias and MM TC classes, respectively. Dunn's test was performed for nonparametric pairwise multiple comparisons between all analyzed groups in PC dyscrasia and MM samples datasets and the Benjamini-Hochberg correction was applied for multiple comparisons. Statistical analysis indicates a significant p-value between N and pPCL (*p* = 0.0211) for *JAG1*; N and pPCL (*p* = 0.0071), MM and pPCL (*p* = 0.0043) for *HES5*; TC5 and the other TC classes (TC1 *p* = 0.0068, TC2 *p* = 0.0215; TC3 *p* = 0.0150; TC4 *p* = 0.0112) for *NOTCH2*; TC5 and TC1(*p* = 0.0056) for *HES5*; TC5 and TC3 (*p* = 0.0239) for *HES6*, respectively.

To investigate transcript expression levels of Notch signaling members, we analyzed highly purified CD138^+^ PC samples from primary MM patients. Gene expression profiling (GEP) analysis carried out in a proprietary data set of 129 MM, 24 primary plasma cell leukemia (pPCL) patients and 4 normal controls revealed that the Notch transcriptional target *HES5* and the Notch ligand *JAG1* are overexpressed in the different types of PC dyscrasias compared to normal controls, reaching a higher expression level in pPCLs (Figure 1A).

In addition, we analyzed the expression patterns of Notch signaling members in 129 MM cases molecularly stratified on the basis of the presence of the main IgH chromosomal translocations and cyclin D expression (TC classification) [50] including 33 TC1, 28 TC2, 38 TC3, 19 TC4 and 6 TC5 cases. Expression of *NOTCH2* was significantly increased in the six TC5 MM patients (Figure 1B), which carry the high-risk translocations t(14;16)(q32;q23) and t(14;20)(q32;q11). Also, two Notch transcriptional target genes, *HES5* and *HES6*, show higher expression levels in TC5 compared to other TC classes. GEP results were validated by qRT-PCR (Figure S1 and Supplemental Information).

Patients affected by pPCL or belonging to the TC5 subgroup are characterized by the presence of highly proliferative and aggressive tumor cells. These malignant cells show a reduced dependency on signals provided by the BM, mainly IL-6, thus prompting us to hypothesize that hyperactive Notch signaling may participate in MM progression by compensating or activating IL-6 proliferative signaling.

### Notch activity is necessary for the acquisition of IL-6 independency

To test the hypothesis that IL-6 independency could occur through cell-autonomous compensatory mechanisms, we analyzed the Notch signaling pathway activity in the human myeloma cell lines (HMCLs) CMA-03 and CMA-03/06, a cell model of MM acquisition of IL-6 independency previously established by our group [51, 52]. This model comprises the CMA-03 parental cells, which rely on IL-6 stimulation for cell proliferation, and CMA-03/06 cells, which are an IL-6 independent and unresponsive variant obtained after 4 months of culture with decreasing concentrations of IL-6. We reasoned that, if the acquisition of IL-6 independency could be due to the possible activation of Notch signaling, then: 1) the progression to IL-6 independency of CMA-03/06 cells should be accompanied by Notch signaling activation and reverted by Notch signaling inhibition; 2) CMA-03 cells may acquire IL-6-independency by upregulating Notch signaling.

**Figure 2:**
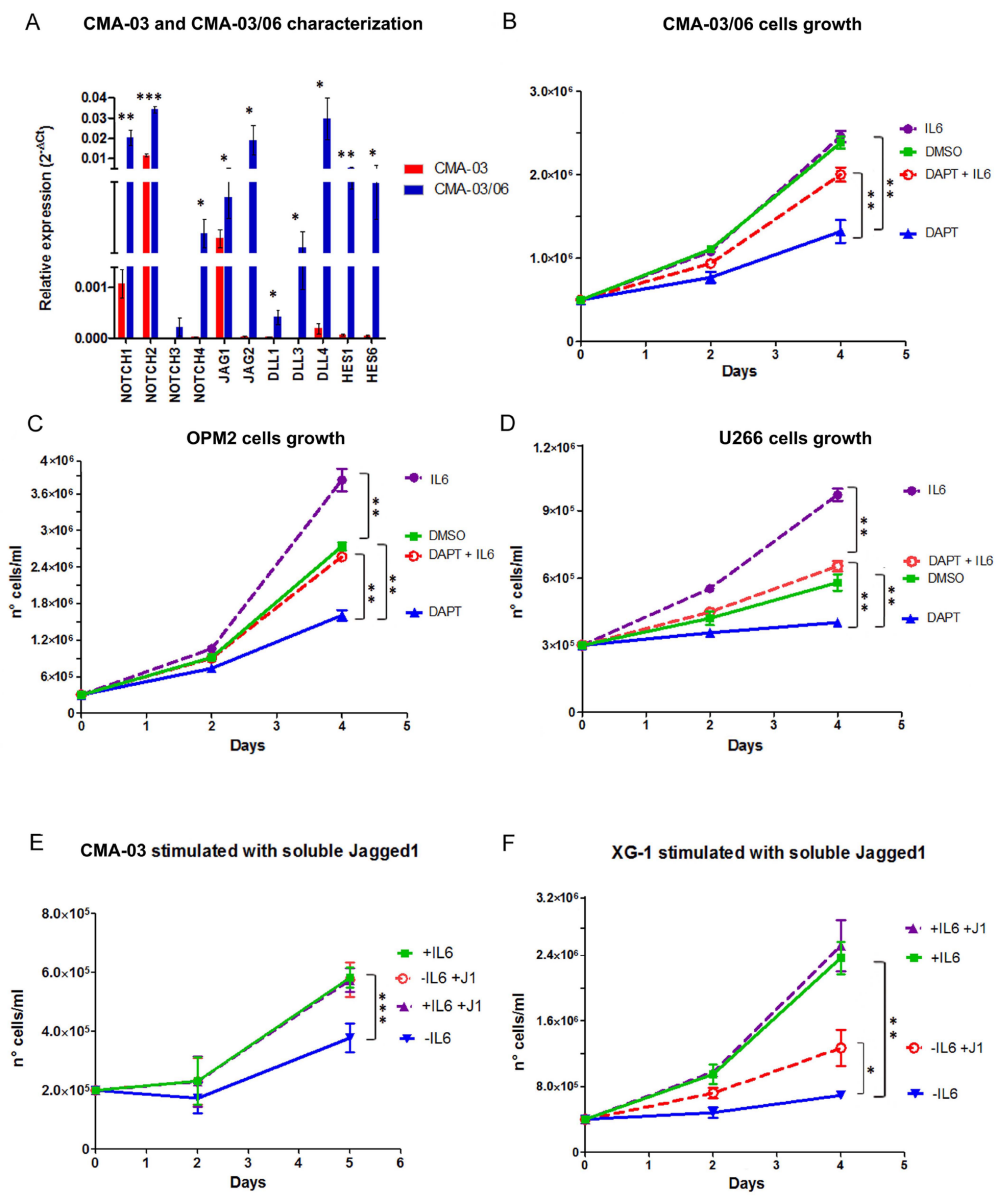
Notch activity reduces the dependency of MM cell lines from IL-6 The contribution of Notch and IL-6 to MM cells growth was evaluated. **A.** The expression levels of Notch receptors (NOTCH1-4), Notch ligands (JAGGED1-2 and DLL1,3,4) and Notch transcriptional target genes, *HES1* and *HES6*, were assessed in the two cell lines, CMA-03 and CMA-03/06, by qRT-PCR. Results were calculated by the 2^−ΔCt^ formula and SDs were calculated from 3 independent experiments. Statistical analysis was performed using Student's t-test: * = *p* < 0.05;** = *p* < 0.01;*** = *p* < 0.001. **B.**-**D.** Cell growth analysis of CMA-03/06 (B), OPM2 (C) and U266 (D) cells treated with or without DAPT 50μM and/or IL-6 for 96h. Mean values ± SD are shown. Statistical analysis was performed using ANOVA and Tukey test: ** = *p* < 0.01. **E.**-**F.** CMA-03 (E) and XG-1 (F) cells were treated with 5μg/ml soluble JAGGED1 ligand (J1) for 4-5 days: J1 stimulation is able to compensate for IL-6 withdrawal. SD were calculated from 3 independent experiments and statistical analysis was performed using ANOVA and Tukey test: * = *p* < 0.05;** = *p* < 0.01;*** = *p* < 0.001.

To address the first issue, we measured by qRT-PCR the relative expression levels of Notch signaling members in CMA-03 and CMA-03/06 cells. As shown in Figure 2A, we found significant differences in the expression levels of Notch receptors (NOTCH1-4), Notch ligands (JAGGED1-2 and DLL1,3,4) and Notch transcriptional target genes, *HES1* and *HES6*, used as a measure of Notch activation, in the two cell lines. In Figure S2 we showed the expression levels of the above reported genes in CMA-03/06 normalized to CMA-03 cells. Increased expression of Notch receptors and ligands in CMA-03/06 cells was associated with considerable increased levels of *HES1* and *HES6* transcripts. We confirmed Notch signaling activation by analyzing the protein expression of the NOTCH2 active form (the most expressed isoform) and the Notch transcriptional target, HES1, in Western blot (Figure S3). These results indicate that the acquisition of IL-6 independent cell growth was associated with a significant increase in Notch pathway activity.

To confirm that Notch signaling was necessary to maintain IL-6 independency, we inhibited Notch activation in IL-6 independent MM cells by means of γ-Secretase inhibitor (i.e. DAPT). To this purpose, CMA-03/06 cells were treated for 96 hours with 50μM DAPT, 10ng/ml IL-6, or a combination of both compounds; the respective vehicles, DMSO and BSA, were used as controls. The DAPT-mediated Notch withdrawal was confirmed by evaluating the expression of the Notch target gene *HES6* by qRT-PCR (Figure S4). Figure 2B shows that DAPT treatment significantly reduced cell proliferation, which could be largely recovered by IL-6 administration. It should be noted that exogenous IL-6 was capable of stimulating CMA-03/06 cell proliferation when Notch signaling is depleted, although it was ineffective when Notch signaling is active. Therefore, CMA-03/06 cells regain both responsiveness and dependency on IL-6 when deprived of Notch proliferative stimuli, indicating that Notch signaling may compensate IL-6 activity. The results obtained in CMA-03/06 cells were confirmed in two other IL-6-independent HMCLs, OPM2 and U266, in which decreased cell proliferation induced by Notch withdrawal (DAPT; Figure S4) was reverted by IL-6 stimulation (Figure 2C-2D).

To verify if Notch signaling was sufficient to induce IL-6 independent cell growth in CMA-03 cells, we activated endogenous Notch signaling by stimulation with soluble JAGGED1 [40, 46]. For this purpose, CMA-03 cells were treated with 5 μg/ml soluble JAGGED1 ligand in the presence or absence of IL-6 (10 μg/ml). Jagged1 stimulation was able to compensate for IL-6 withdrawal with a complete recovery of cell growth (Figure 2E). This finding was confirmed in another IL-6-dependent HMCL, XG-1 (Figure 2F; Figure S5). In this case, JAGGED1-mediated Notch stimulation recovered 45% of cell growth in the absence of IL-6, indicating that Notch signaling can compensate for the lack of IL-6 proliferative stimulus in IL-6 dependent MM cell lines.

### Notch signaling promotes MM cell-autonomous IL-6 production

**Figure 3:**
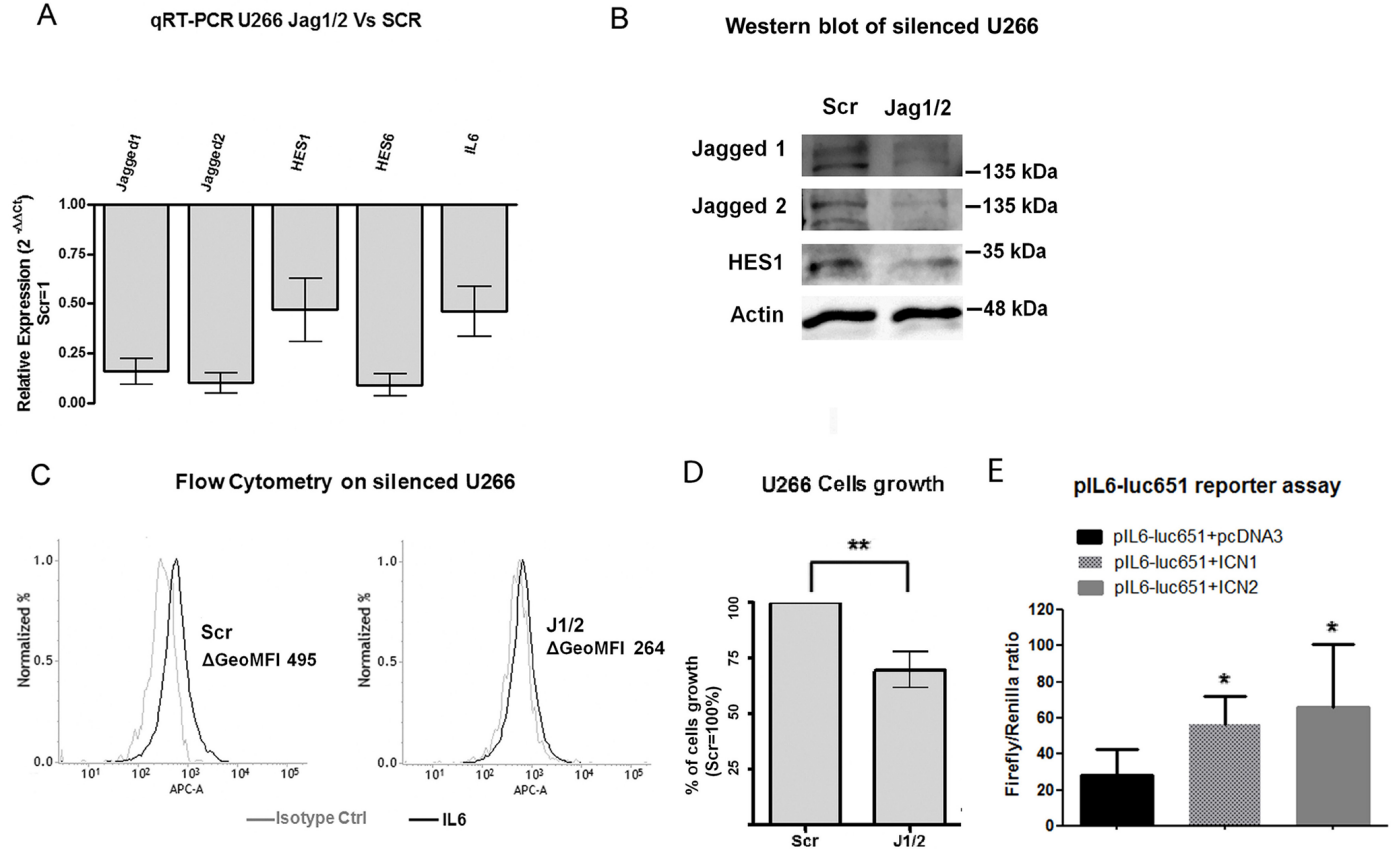
Notch drives MM cell-autonomous production of IL-6 and stimulates their proliferation U266 cells were transfected with two specific siRNAs targeting *JAGGED1* and *JAGGED2* (J1/2) or the corresponding scrambled control (Scr). **A.** Confirmation of J1/2 silencing efficiency in U266 cells was obtained by qPCR measurements of relative gene expression variation of JAGGED1 and JAGGED2 and Notch target genes *HES1* and *HES6* in cells transfected with siRNA against J1/2 compared to cells transfected with scrambled control, calculated by the 2^−ΔΔCt^ formula. *IL-6* expression levels were also analyzed. SDs were calculated from 3 independent experiments. Two-tailed t-test confirmed statistically significant downregulation of the genes tested; **B.** Western blot confirmed that JAG1, JAG2 and HES1 are downregulated in silenced U266; **C.** Histograms display the levels of intracellular IL-6 analyzed by flow cytometry in U266-J1/2kd or U266-Scr in single culture (black lines), the isotype matched control is shown in gray. ΔGeoMFI were obtained by subtracting the appropriate isotype control from the positive signal. Histograms are representative of 3 experiments with similar results. **D.** J1/2 silencing in U266 cells caused a significant decrease in cell proliferation. Mean values ± SD are shown. Statistical analysis was performed using two-tailed t-test: ** = *p* < 0.01; **E.** Luciferase reporter assay showing that both ICN1 and ICN2 are able to boost the activity of IL6 promoter. Mean values ± SD are shown. Statistical analysis was performed using one-tailed, unpaired *t*-test * = *p* < 0.05.

To investigate whether Notch signaling may promote endogenous *IL-6* expression, we took advantage of U266 cell line, a cellular model of MM characterized by IL-6 independency due to autocrine production of IL-6 [53]. We used a previously validated genetic Notch inhibitory approach based on the knockdown of *JAGGED1* and *2* (J1/2) ligands [46]. The efficacy of *J1/2* silencing was measured by assessing the decrease in *JAG1, JAG2*, *HES1* and *HES6* gene expression levels by qRT-PCR (Figure 3A) and JAG1, JAG2 and HES1 protein level by western blot (Figure 3B). qRT-PCR and flow cytometry demonstrated that *J1/J2* silencing reduced both *IL-6* transcript and protein level by approximately 50% (Figure 3A and Figure 3C). For flow cytometric measures the statistical significance was calculated on the value of geometric mean fluorescent intensity subtracted of the appropriate isotype control (ΔGeoMFI) of 3 independent experiments (Figure S6). As expected, Notch withdrawal through *J1/2* knockdown inhibited U266 cell growth by approximately 30% (Figure 3D), due to an increase in the apoptotic rate and to a partial cell cycle arrest in G0/G1 phase (Figure S7 and Supplemental Information).

We demonstrated that *IL-6* is a Notch transcriptional target in MM cells by a dual luciferase assay in U266 cell line using an *IL-6* reporter (pIL6-luc651) transactivated by plasmids carrying the active forms of NOTCH1 (ICN1) and NOTCH2 (ICN2). Results, reported in Figure 3E, clearly showed that ICN1 and ICN2 can transactivate the *IL-6* responsive element.

To investigate the existence of a direct correlation between Notch and IL-6 in primary cells from MM patients, we analyzed microarray gene expression data (GEO dataset No. GSE66293). The analysis on the whole dataset failed to identify a statistically significant correlation between *IL-6* and *HES6* (data not shown). When patients were stratified according to *IL-6* expression levels, the results obtained on patients showing the highest expression of *IL-6* by microarray analysis (Figure S8-A) and qRT-PCR (Figure S8-B) indicated positive, even if not statistically significant, Pearson's correlation indexes (*r* = 0.54 and *r* = 0.71; *p* = 0.1358 and *p* = 0.0573, respectively), possibly due to the small sample size.

### Notch signaling promotes BMSC pro-tumor effect by stimulating paracrine IL-6 production

BMSCs are the most important source of IL-6 in MM [10, 11]. Therefore, we verified if MM cell-derived Jagged ligands could trigger Notch activation in BMSCs [49] and boost IL-6 production.

**Figure 4:**
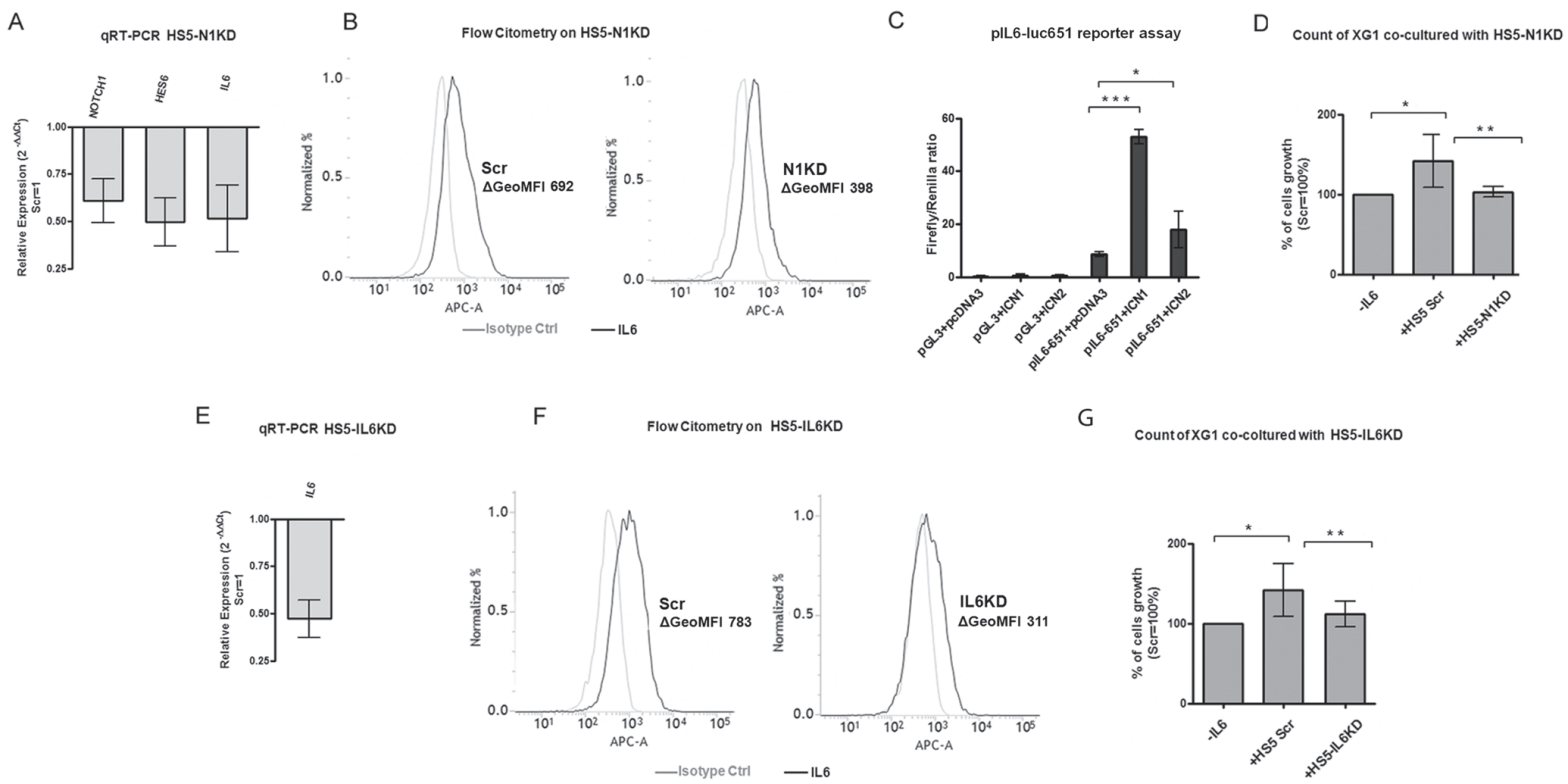
BMSCs produce IL-6 and support MM cell growth in a Notch-dependent manner We evluated the contribution of the Notch pathway on the ability of stromal cells to support MM cell growth. **A.** Confirmation of NOTCH1 inhibition in HS5-N1kd by qPCR measurements of *NOTCH1, HES6* and *IL-6* gene expression. HS5-N1kd cells were compared to HS5-Scr control cells. Fold changes were calculated by the 2^−ΔΔCt^ formula as reported above. SD were calculated from 3 independent experiments. **B.** Histograms display the levels of intracellular IL-6 (black line) analyzed by flow cytometry in HS5-Scr or HS5-N1kd cells, and an isotype-matched control (gray line). ΔGeoMFI were obtained by subtracting the appropriate isotype control from the positive signal. Histograms are representative of 4 independent experiments with similar results. **C.** Luciferase assay on the stromal-mimetic cell line NIH3T3 showed that both ICN1 and ICN2 promotes the activation of IL6 promoter. Mean values ± SD are shown. Statistical analysis was performed using one-way ANOVA and Tukey test: * = *p* < 0.05; *** = p < 0.001. **D.** Cell growth analysis of XG-1 cells cultured alone or co-cultured with HS5-Scr or HS5-N1kd. Mean values ± SD are shown. Statistical analysis was performed using one-way ANOVA and Tukey test: * = *p* < 0.05, ** = *p* < 0.01. **E.** Confirmation of *IL-6* silencing was obtained by qPCR of *IL-6* gene expression; fold change was calculated by the 2^−ΔΔCt^ formula as reported above. SD was calculated from 3 independent experiments. **F.** Histograms displayed the levels of intracellular IL-6 (black line) analyzed by flow cytometry in HS5-Scr or HS5-IL6kd, and an isotype-matched control (gray line). ΔGeoMFI were obtained by subtracting the appropriate isotype control from the positive signal. Histograms are representative of 5 independent experiments with similar results. **G.** Cell growth analysis of XG-1 cells cultured alone or co-cultured with HS5-Scr or HS5-IL6kd. Mean values ± SD are shown. Statistical analysis was performed using one-way ANOVA and Tukey test: * = *p* < 0.05, ** = *p* < 0.01.

First, we verified whether *IL-6* expression was under Notch signaling control in BMSCs. To test this hypothesis, Notch signaling was inhibited in the human BMSC line HS5 by transfecting NOTCH1-targeting siRNA (HS5-N1kd). After 96 hours, Notch pathway inhibition in HS5-N1kd cells was verified by qRT-PCR and analysis of *HES6* gene expression (Figure 4A). HS5-N1kd cells showed a significantly reduction in *IL-6* gene expression (Figure 4A), which was confirmed at the protein level by flow cytometry (Figure 4B). We confirmed Notch ability to transactivate *IL-6* promoter in BMSCs by a dual luciferase assay in NIH3T3 cell line, a murine easy transfectable mimic of BMSCs (Figure 4C). Then, we determined whether the Notch-deprived BMSCs maintained the ability to support the proliferation of the IL-6-dependent cell line XG-1. For this purpose, XG-1 cells were cultured for 48h on a layer of GFP^+^ HS5 cells transfected with siRNA targeting Notch1 or with the scrambled (Scr) control. XG-1 cells were enumerated by absolute counts of GFP^−^ cells by flow cytometry. Results shown in Figure 4D indicated that HS5-N1kd cells were no longer able to support XG-1 cell growth that was comparable to the absence of IL-6. This effect was mainly due to increase of tumor cells undergoing apoptosis, while no significant cell cycle changes were observed (Figure S9).

To confirm that the lack of IL-6 production in HS5-N1kd cells was responsible for their impaired ability to support XG-1 cell proliferation, we knocked down *IL-6* expression in HS5 (HS5-IL6kd) by transfecting them with anti-*IL-6* siRNA, and co-cultured these or control cells (HS5-SCR) with XG-1. *IL-6*-silencing in HS5 cells, confirmed at the mRNA and protein level by qRT-PCR and flow cytometry, respectively (Figure 4E and 4F), resulted in a significant decrease in cell growth of co-cultured XG-1 (Figure 4G).

### MM cell-derived JAGGED ligands activate Notch signaling in BMSCs, promoting IL-6 production and tumor growth

**Figure 5:**
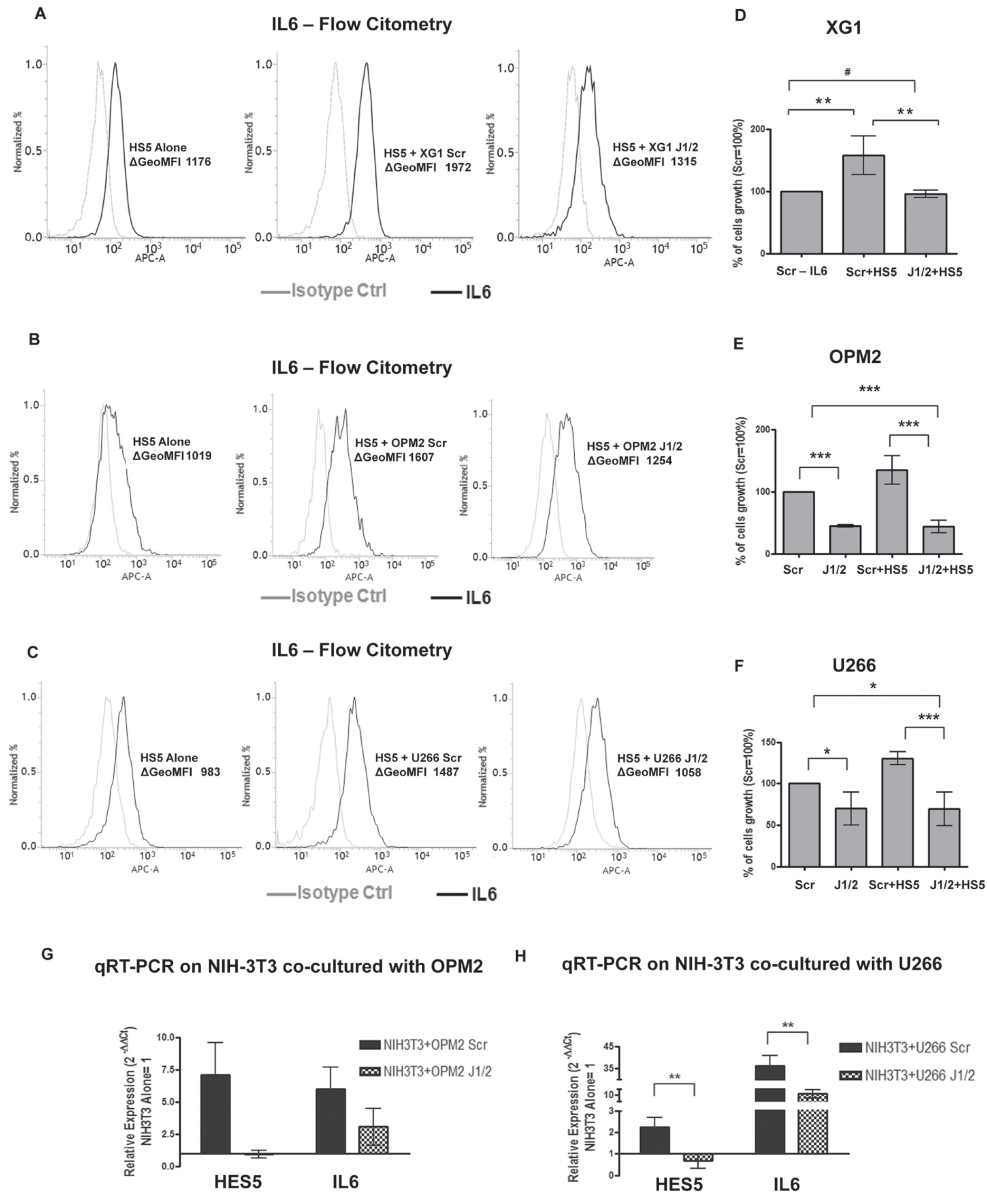
MM-derived JAGGED promotes IL-6 production by BMSCs MM cells are able to shape the BM microenvironment and increase IL-6 production. We established co-cultures of MM cells with the BM stromal cell line HS5. Histograms display the levels of intracellular IL-6 (black lines) analyzed by flow cytometry in HS5 cells in single culture or after co-culture with XG-1-Scr or XG-1-J1/2kd (**A**), OPM2-Scr or OPM2-J1/2kd (**B**) and U266-Scr or U266-J1/2kd (**C**) cells, and an isotype-matched control (gray line). ΔGeoMFI were obtained by subtracting the appropriate isotype control from the positive signal. Histograms are representative of 3 independent experiments for XG-1, 3 for OPM2 and 4 for U266 cells, with similar results. **D.**-**F.** Cell growth analysis of HMCLs-J1/2kd or HMCLs-Scr cultured alone or co-cultured with HS5 cells, specifically XG-1 (D), OPM2 (E) and U266 cells (F) Mean values ± SD are shown. Statistical analysis was performed using one-way ANOVA and Tukey test: * = *p* < 0.05;** = *p* < 0.01; *** = *p* < 0.001. **G.**-**H.** qRT-PCR for *IL-6* and *HES5* gene expression in NIH3T3 cells co-cultured with OPM2-J1/2kd or OPM2-Scr (G) or U266-J1/2kd or U266-Scr (H) compared NIH3T3 cultured alone (= 1), calculated by the 2^−ΔΔCt^ formula. *HES5* was used as a control for pathway activity. Mean values ± SD are shown. Statistical analysis was performed using two-tailed *t*-test: ** = *p* < 0.01; *** = *p* < 0.001.

We investigated the possible contribution of dysregulated Notch ligands in influencing the ability of MM cells to shape the BM microenvironment. The expression of JAGGED1 and 2 was inhibited in IL-6-sensitive HMCLs (XG-1, OPM2 and U266 cells) by transfection with J1/2 siRNAs or a SCR control (HMCLs-J1/2kd or HMCLs-SCR). Transfected MM cell lines were co-cultured with GFP^+^ HS5 to distinguish the two cell types. Flow cytometry analyses demonstrated that the percentage of IL-6 produced by HS5 cells was increased of about the 40-50% when cultured in the presence of HMCLs-SCR, but this increase was markedly reversed when HS5 were cultured with HMCLs-J1/2kd (Figure 5A-5C; Figure S10). HS5 cells co-cultured with HMCLs-J1/2kd were no longer able to support tumor cell growth (Figure 5D-5F). We analyzed the outcome on XG-1 cells growth since they display a complete dependence on the support of HS5 or IL-6 and therefore they represent a reliable cell model for MM. Results indicated that reduction of XG-1 cell number was due to apoptosis (Figure S12).

We also demonstrated that the observed changes in IL-6 protein levels reflected the variations in *IL-6* gene expression by using co-culture systems of human OPM2 or U266 cells and murine fibroblasts NIH3T3 that allowed us to identify the origin of mRNAs by RT-PCR analysis with species-specific primers. Results showed that the Notch pathway was activated as demonstrated by increased murine *hes5* gene expression (Figure 5G-5H) and Notch activation was accompanied by an increase in murine *il-6* gene expression. As expected, *JAGGED1-2* silencing impaired the ability of MM cells to activate the Notch pathway in stromal cells, causing a decrease in the production of *il-6* (Figure 5G-5H).

### Notch signaling withdrawal reduces the release of IL-6 in co-cultures of primary MM cells and BMSCs

**Figure 6:**
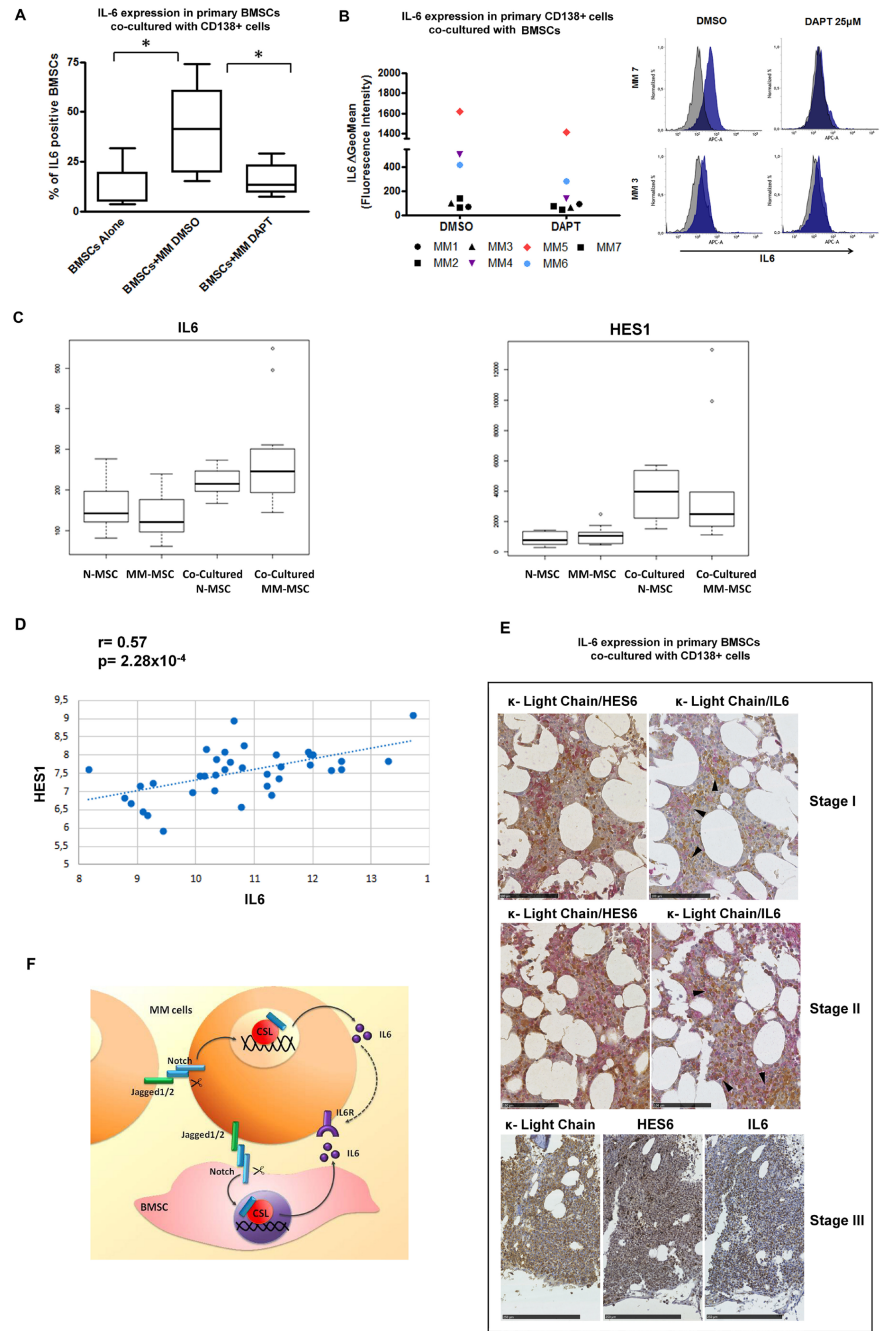
Inhibition of NOTCH signaling abrogates myeloma-induced IL-6 production by BMSCs *ex-vivo* **A.** Levels of intracellular IL-6 were analyzed by flow cytometry in primary BMSCs co-cultured with CD138^+^ cells from MM patients in the presence or absence of 25μM DAPT. Seven independent experiments were performed and the percentage of IL-6^+^ BMSCs is shown in the bar graph. Statistical analysis was performed using one-way ANOVA and Bonferroni post-test. (* = *p* < 0.05). **B.** Analysis of intracellular IL-6 levels by flow cytometry in primary CD138^+^ cells co-cultured with BMSCs from MM patients in the presence/absence of 25 μM DAPT. Scatter plot shows the ΔGeoMFI of all the analyzed IL-6^+^ (colored dots) and IL-6^−^ (black dots) samples (left). Rapresentative histograms of IL-6^+^ and IL-6^−^ MM primary cells are also showed (right). **C.** Gene expression profiles of *IL-6* and *HES1* in 37 mesenchymal stromal cell (MSC) samples from 16 healthy donors and 21 MM cases, alone (8 N-MSC, 9 MM-MSC) or in co-culture with MM1S cell line (8 N-MSC, 12 MM-MSC), extracted from an external database (GSE46053) profiled on Affymetrix HG-U133 Plus 2.0 array. Kruskal-Wallis test was performed to assess statistical significance of differential gene expression profiles between all four MSC groups. Dunn's test and the Benjamini-Hochberg correction were applied for nonparametric pairwise multiple comparisons. Statistical analysis indicates a significant *p*-value for *IL-6* in N-MSC and MM-MSC alone groups compared to both co-cultured N-MSC and MM-MSC samples (*p* = 0.0008, *p* = 0.0018 for N-MSC alone; *p* = 0.0012, *p* = 0.0038 for MM-MSC alone), respectively; similarly for *HES1* in N-MSC and MM-MSC alone compared to both co-cultured N and MM groups, respectively (*p* = 0.0485, *p* = 0.0101 for N-MSC alone; *p* = 0.0135, *p* = 0.0017 for MM-MSC alone). **D.** Pearson's correlation plots of *IL-6* and *HES1* expression levels (log2 scale) in the same data set. **E.** *Representative images* of *IHC* staining for HES6 and IL-6 (brown immunoreactivity) in human BOM infiltration by κ light chain myeloma (MM) (red immunostaining) at different stages (for each tumor stage, consecutive histological sections were stained with different; bars markers represent 100μm for stage I and II and 250μm for stage III) antigens) Stage I MM: HES6 immunoreactivity can be easily detected both in neoplastic and in stromal cells; IL-6 immunostaining is detectable in non-neoplastic cells surrounding myelomatous nests (arrows). Stage II MM: A more intense HES6 immunoreactivity can be detected in both BM components; IL-6 immunoreactivity can be observed also in some MM cells. Stage III MM: in late stages of tumor infiltration, intense HES6 and IL-6 immunoreactivity is appreciable in κ light chain immunoreactive MM cells. In this case, to better visualize HES6 and IL-6 immunoreactivity and distribution, single antigens immunoreactivity was evaluated individually on each consecutive slide; the same BM areas of the case under study are here compared. **F.** Schematic illustration of the JAGGED-directed mechanisms of autocrine and paracrine IL-6 expression in MM.

We verified whether the ability of BMSCs to release IL-6 was dependent on MM cell-mediated Notch signaling activation by using primary co-culture systems of highly purified CD138^+^ MM cells and BMSCs from 7 MM patients' BM aspirates, purified as described by Garayoa *et al*. [54]. Co-cultures were maintained for 96 hours in the presence or absence of 25 μM DAPT and changes in BMSC-mediated IL-6 production were measured by flow cytometry. These analyses showed that primary MM cells significantly increased the fraction of IL-6-producing BMSCs (from 12% to 41% IL-6^+^ BMSCs, Figure 6A). In contrast, DAPT-mediated Notch inhibition significantly reduced the proportion of IL-6-producing BMSCs (15% IL-6^+^ BMSCs, Figure 6A), confirming our *in vitro* findings in HS5/HMCL co-cultures. Concerning MM-derived IL-6, since primary MM cells do not survive without BMSCs or IL-6 stimulation, the evaluation of possible variations in IL-6 production by MM cells in the absence of BMSCs was not possible. Nevertheless, flow cytometry detection of intra-cellular IL-6 allowed us to measure the number of IL-6 positive MM cells when they were cultured in the presence of BMSCs. We could observe that CD138^+^ MM cells from 3 out of 7 patients expressed appreciable levels of IL-6 (ΔGeoMFI >400, Figure 6B). Furthermore, co-cultured MM cells treated with 25 μM DAPT displayed considerably reduced levels of IL-6 expression in each of the three IL-6-positive MM patient samples, with a median decrease of about 40% (Figure 6B).

The ability of MM cells to boost IL-6 production from BMSC was further confirmed by analysing the correlation between IL-6 and HES1 gene expression in the dataset of Garcia Gomez et al. [55] including 37 mesenchymal cell samples from healthy donors or MM patients, cultured alone or co-cultured with the MM1S cell line. As shown in Figure 6C and 6D, these results confirmed a positive correlation between the expression of IL-6 and HES1 (*r* = 0.57 and *p* = 2.28×10-4) and showed IL-6 and HES1 gene expression upregulation upon stimulation of mesenchymal cells with MM1S myeloma cells.

Finally, we investigated whether there was a correlation between Notch activity and IL-6 in the endogenous MM BM niche by analyzing BM biopsies from 21 MM patients. Immunoreactivity of IL-6 and HES6 was assessed by immunohistochemistry (Figure 6C). In all cases investigated, HES6 immunoreactivity was detected both in malignant PCs and in surrounding non-myelomatous cells. Moreover, the intensity of the immunoreactive signal increased with MM infiltration in the BM. IL-6 immunoreactivity is gradually acquired by MM cells during BM invasion in neoplastic progression. In the early stage of infiltration by MM cells, IL-6 immunoreactivity was almost exclusively localized in non-myelomatous cells, surrounded by small groups of MM cells (Figure 6C). Rare MM cells with faint cytoplasmic IL-6 immunoreactivity could be detected in this stage. IL-6 immunopositive neoplastic cells increased in intermediate stages of BM infiltration (up to 30% MM cells), and were mainly localized near the bone spicule. In late stages, most MM cells were positive for IL-6, the IL-6 negative cells being no more than 5%.

No significant differences in IL-6 immunoreactivity were observed in positive cells of all investigated cases.Overall, these results suggest a novel Notch-dependent mechanism by which the BM microenvironment may support autocrine and paracrine IL-6 production thereby promoting MM cell growth (Figure 6D).

## DISCUSSION

MM is the second among hematological malignancies and affects about 600,000 people every year in Europe and the USA [56]. Despite the introduction of several new treatments, improving the clinical course of disease, myeloma remains an incurable cancer with a survival rate of 45% at 5 years [57]. The Notch pathway has been proposed as a possible therapeutic target in MM [41] due to its role in key aspects of MM, i.e. drug resistance [42–44], stemness [45], bone disease [46, 47] and migration to BM [49].

The aim of the present study was to deepen the understanding of the role of Notch signaling upregulation [36–40] in MM by investigating its cross talk with the chemokine IL-6, a key player of MM progression and severity [15–19]. Our first observation was obtained by GEP analysis and indicated that the trend of Notch signaling activation (evaluated by *HES5* expression levels) reflected the acquisition of a highly malignant phenotype, since it occurred in high-risk cytogenetics MM and PCL patients who displayed the highest activation levels. A similar trend is associated with gene expression levels of the Notch ligand *JAGGED1*, consistent with the immunohistochemistry studies by Skrtic and coworkers on JAGGED1 protein [38] and confirming the key role played by this ligand in Notch autonomous activation as we previously reported [46]. The stratification of MM patients according to the TC molecular classification [50] indicated an association of the MAF-translocated group with the highest *NOTCH2* expression levels and increased Notch activity (*HES5* and *HES6* expression levels). This was in agreement with the ability of C-*MAF* and *MAFB* to activate Notch signaling by transactivating *NOTCH2* gene expression [58]. Significantly, also MAF translocations are frequently associated with high-risk PCLs or aggressive forms of *de novo* MM without preceding MGUS [50, 59].

Overall, these results indicate that the activation of Notch signaling characterizes the most aggressive form of MM and is associated with high-risk disease onset or evolution.

Since highly malignant MM cells show a reduced dependency on signals provided by the BM, at least in part due to the cell-autonomous production of IL-6 [6, 8], we wondered if the upregulation of Notch signaling could play a role in promoting the malignant progression of MM cell by reducing the dependency on signals provided by BM such as IL-6.

This hypothesis was verified in our CMA-03 model of MM progression, demonstrating that: 1) the acquisition of IL-6 independent cell growth was associated with the upregulated expression of Notch receptors and ligands together with increased transcription of Notch target genes; 2) when stimulated with soluble JAGGED1 ligands, IL-6-dependent CMA-03 cells acquired the ability to grow in the absence of IL-6; and 3) upon DAPT administration, IL-6-independent and unresponsive CMA-03/06 cells recovered IL-6 dependency. The general validity of these findings was confirmed on other HMCLs and prompted us to postulate that clonal selection leading to the development of IL-6-independent MM clones might involve the upregulation of the Notch pathway. Thereby, these results indicate a novel pathogenic role of Notch signaling activation in MM cell consisting in inducing the acquisition of IL-6 independency.

By luciferase reporter assay we demonstrated that in MM cells able to autonomously produce IL-6, such as the U266 cell line [9, 13], the acquisition of IL-6 independency may be due to Notch ability to transactivate *IL-6* promoter. Indeed, in U266 cells Notch activity was necessary to maintain the autonomous IL-6 expression, that significantly decreased upon Notch withdrawal induced by RNA interference of JAGGED ligands. These *in vitro* results were confirmed in primary cells from three out of seven patients autonomously expressing IL-6 (36% IL-6 positive cells on average), whose expression declined upon treatment with DAPT (14% IL-6 positive on average). Further confirmation came from immunohistochemical analysis of BM biopsies obtained from MM patients. In particular, we observed that the ability of MM cells to produce IL-6 was acquired along disease progression through the different stages of BM infiltration and was associated with increased Notch signaling activation in MM cells.

Finally, a correlation analysis of gene expression levels in primary cells from MM patients, obtained by GEP analysis and RT-PCR, suggested that only primary MM cells of patients with the higher levels of *IL-6* show a direct correlation between Notch activity (*HES6* gene expression) and *IL-6* expression. However, these results did not reach the statistical significance possibly due to the limited sample size and or the effect of other inflammatory stimuli from BM, i.e. NF-κB, affecting *IL-6* expression in primary MM cells. Indeed, according to Wongchana and Palaga, Notch signaling is subordinated to NF-κB in the activation of *IL-6* transcription [60]. A full elucidation of the interplay between Notch and BM-derived inflammatory stimuli in IL-6 production occurring in MM cells is needed.

Overall, these findings support the notion that Notch may induce the independency from BM niche and IL-6 stimulus by promoting the autocrine IL-6 production in MM cells, thereby contributing to progression to the extramedullary phase. We anticipate that other mechanisms of cooperation between the IL-6 and Notch pathways may exist since Notch activation may compensate IL-6 proliferative signal even in MM cells that do not autonomously produce IL-6 (i.e. OPM2, CMA-03/06, XG-1).

Beside the possible tumor autonomous production of IL-6, BMSCs represent the main source of IL-6 for myeloma cells [10–12], therefore we examined whether Notch signaling could affect the release of IL-6 from BMSCs. Our results showed that both ICN1 and ICN2 are able to transactivate *IL-6* promoter in the NIH3T3 murine fibroblast cell line used as mimic of BMSCs. Moreover, in human BMSCs Notch activity was necessary for the production of IL-6 protein; indeed, RNA interference of *NOTCH1* resulted in decreased *IL-6* mRNA and protein expression in the human line HS5. Accordingly, HS5-N1kd cells have a reduced ability to support the growth of IL-6-dependent XG-1 cell line.

We investigated if MM cells were able to activate Notch signaling in BMSCs through the overexpressed JAGGED ligands. Indeed, we observed that HMCLs were able to boost the amount of IL-6 produced by human (HS5) or murine (NIH3T3) BMSCs. This effect was dependent on the expression of JAGGED ligands, since the increase in BMSC-derived IL-6 production and BMSC support to tumor cell growth were completely abrogated upon depletion of JAGGED ligands in the co-cultured HMCLs. Coherently, Houde *et al*. stimulated IL-6 production from the MRC5 lung fibroblast cell line by using synthetic JAGGED peptides [40] and by Sethi and coworkers demonstrated that breast cancer cell-derived JAGGED induced IL-6 production by BMSCs [61].

By *ex vivo* co-culture systems, we further confirmed the ability of CD138^+^ MM cells to stimulate primary BMSCs from MM patients to produce IL-6, as well as Notch involvement since the stimulation was abrogated by treatment with γ-Secretase inhibitor.

These results were confirmed by a the evidence of a correlation between the expression of IL6 and Notch activation in immunohistochemical studies and GEP assays. Indeed analyses on BM biopsies from MM patients indicated that in early stages of disease, IL-6 immunoreactivity was detectable only in discrete non-myelomatous cells that expressed IL-6 only when in proximity to MM cells. Similarly, Notch activation was detected in malignant PCs and in surrounding non-myelomatous cells. Finally, GEP analysis on mesenchymal stromal cells from healthy volunteers and MM patients of Garcia Gomez *et al*. dataset [55] confirmed a correlation between the expression of *IL-6* and *HES1* and additionally showed *IL-6* and *HES1* upregulation upon stimulation with the MM1S cell line.

Ovearll, these data attribute to Notch a function in MM pathogenesis as a key signaling pathway in tumor-stroma communication exploited by MM cells to shape the BM niche and specifically to increase the release of a key cytokine such as IL-6.

In conclusion, this work offers novel insights into the role of Notch signaling in MM, providing the first experimental evidence that MM-derived JAGGED ligands drives IL-6 production by MM cells and surrounding BM niche (Figure 6D). Together, these findings provide a new paradigm where high-risk MM/pPCL is characterized by high levels of Notch signaling that can partially compensate for lower IL-6 in the extramedullary microenvironment, possibly by activating IL-6 autonomous production. On the other side, MM cells residing in the BM, are still dependent on IL-6, but can sustain their own progression by stimulating BMSC-mediated IL-6 production through JAGGED ligands. The present findings strengthen the rationale for Notch-tailored therapies in MM providing evidences that knocking down MM-derived JAGGED ligands may reduce MM tumor burden by decreasing the amount of IL-6 in the BM niche.

## MATERIALS AND METHODS

### Gene expression profiling

Highly purified PC samples (CD138 ≥ 90%) from the BM of 129 MM and 24 primary plasma cell leukemia (pPCL) patients at onset, together with 4 healthy donors (N) samples, were previously profiled on the GeneChip Human Gene 1.0 ST array (Affymetrix, Santa Clara, CA) [62]. Main molecular genomic aberrations (IGH translocations, hyperdiploidy, del(13q), del(17p), and 1q gain) were investigated in all samples by fluorescence in situ hybridization (FISH), as previously described [63] and already reported for the entire sample dataset [62]. Multiple myeloma samples were stratified in five groups according to the translocation/cyclin D (TC) classification [50, 64]. Gene expression profiling data were generated as described [65], using Brainarray annotation procedure [37, 66]. The GEP data have been deposited in the NCBI Gene Expression Omnibus database (GEO; http//www.ncbi.nlm.nih.gov/geo; accession No. GSE66293, GSE73452). The Institutional Review Board of Fondazione IRCCS Policlinico Ca' Granda, Milano, Italy, approved the design of this study. Written informed consent was obtained in accordance with the Declaration of Helsinki.

### Cells and treatments

The human MM cell lines XG-1, CMA-03, CMA-03/06, U266 and OPM2 were cultured in RPMI1640 with 10% FBS, and for XG-1 cells and CMA-03 cells 1ng/ml and 10ng/ml IL-6 were added, respectively. NIH3T3 and human bone marrow stromal (HS5) cell lines were cultured in Dulbecco's modified Eagle's medium (DMEM) with 10% FBS. The γ-secretase inhibitor, DAPT (Sigma-Aldrich, Germany), was reconstitution in DMSO and prepared at a final concentration of 25-50 μM. Recombinant human IL-6 (R&D Systems, USA) was resuspended in PBS supplemented with 0,1% w/v BSA and used at 10ng/ml. Primary cells were isolated from primary patient BM aspirates and MM cells were purified using the Human Whole Blood CD138^+^ Selection Kit EasySep (StemCell Technologies). Primary BMSCs were isolated as previously reported by Garayoa et al [54]. Briefly, mononuclear cells from BM aspirates were obtained after density gradient centrifugation using Ficoll-Paque Premium 1.073 (Sigma Aldrich, USA) and cultured in DMEM supplemented with 10% heat-inactivated FBS for 3-4 days. Subsequently non-adherent cells were removed, whereas stromal cells were selected by their adherence to plasticware.

### Establishment of stable GFP^+^ HS5 cells

The pGIPZ vector (Thermo Scientific) was used to stably transduce HS5 cells in order to obtain a GFP-positive HS5 cell line (GFP^+^ HS5). The production of lentiviral supernatant was performed following the manufacturer's instructions. For transduction, 3×10^5^ HS5 cells were plated in a 6-well tissue culture plate with 1ml of lentiviral supernatant and 4μg/ml of polybrene (Sigma-Aldrich). After the infection cells were selected using 1μg/ml puromycin (Sigma-Aldrich).

### Co-cultures of MM cells and BMSCs

For co-culture experiments, MM cell lines were seeded in a 24-well plate at a density of 3×10^5^ cells/ml on a monolayer of ^+^ or NIH3T3 cells (70-80% confluence) and cultured for 2 days. For primary cell co-cultures, BMSCs, isolated as described above, were stained with the fluorescent dye PKH26 (Sigma-Aldrich) and allowed to adhere for 3 hours. CD138^+^ cells were seeded on the monolayer of PKH^+^ BMSCs (50-60% confluence) and cultured for 4 days in the presence/absence of DAPT.

### qRT-PCR

Total RNA was isolated using the miRneasy kit (Qiagen) and cDNA was prepared using RevertAid M-MuLV Reverse Transcriptase (ThermoScientific) [67]. Quantitative PCR (qPCR) was performed as previously described [49, 68]. Primer sequences are reported in Table 1 (Supplementary Information).

### Western blot

Total cells protein were extracted with RIPA lysis buffer as previously reported [69]. Protein samples (40 μg) were run on a 4-12% NuPAGE gel (ThermoScientific), transferred onto a nitrocellulose membrane (Biorad), and blocked with 5% non-fat milk in TBS-T (20 mM Tris-Cl, pH 7.5, 150 mM NaCl, 0.05% Tween-20). Membranes were incubated overnight at 4 °C with rabbit anti-NOTCH2 (1:1000, Cell Signaling), rabbit anti-HES1 (1:1000, Abcam) or rabbit anti-β-actin (1:5000, Sigma), and then with the appropriated HRP-conjugated species-specific secondary antibody (Pierce Protein Biology). Detection was performed by ECL (Biorad) according to the manufacturer's instructions. The signal was detected and analyzed by ChemiDoc MP System (Biorad).

### RNAi assay

Stealth RNAi^TM^ siRNA system (Invitrogen) consisting of small interfering RNA (siRNA) molecules targeting *NOTCH1* (CCGCCUUUGUGCUUCUGUUCUUCGU, ACGAAGAACAGAAGCACAAAGGCGG), *IL-6* (UACAUCUUUGGAAUCUUCUCCUGGG, CCCAGGAGAAGAUUCCAAAGAUGUA) and a scrambled control were used according to the manufacturer's guidelines. The two siRNAs targeting *JAGGED1* and *JAGGED2* were used as previously reported [46]. Transfections were performed for 48-96 hours with 50 nM siRNAs/scrambled control using lipofectamine RNAiMAX (Invitrogen).

### Luciferase reporter assay

The pIL6-651 plasmid carrying *IL-6* promoter [70, 71] was a kind gift of Dr. Norifumi Takeda. The thymidine kinase promoter-driven Renilla luciferase (TK-pRL; Promega Italia s.r.l., Milano, Italy) was used for normalization; pcDNA3.1 mock plasmid was from Invitrogen (Invitrogen Life Technologies Italia, Italy). The plasmids carrying ICN1 and ICN2 were as previously described [72, 73]. Ten micrograms of total DNA were used for electroporation in U266 cells as previously reported [49]; ten micrograms of total DNA were transfected in NIH3T3 cells using TurboFect Transfection Reagents (Thermo Fisher Scientific).

All analyses were performed 48h after transfection using Dual-Luciferase Reporter Assay System (Promega Italia s.r.l., Milano, Italy) according to the manufacturer's directions.

### Flow cytometry

Absolute cell counts were determined using the volumetric count tool of the BD FACSVerse™ System (BD Biosciences, USA). For evaluation of IL-6 expression, cells were fixed with 4% formaldehyde, permeabilized in 0.5% saponin and stained with anti-human IL-6 antibody (eBioscience Inc, USA) or isotype matched control. Cells were processed using the BD FACSVerse™ System (BD Biosciences). For every sample it was measured the geometric mean fluorescence intensity subtracted of the appropriate isotype control (ΔGeoMFI).

### Immunohistochemistry (IHC)

Archival BM biopsies were analyzed from 21 MM patients diagnosed at the Unit of Pathology, A.O. San Paolo, Department of Health Sciences, University of Milan, Milan, Italy. BM biopsies were fixed in 10% neutral buffered formalin, decalcified, routinely processed for conventional histology, and stained with haematoxylin and eosin, and Giemsa and Gomori silver impregnation techniques. All cases under study had a κ light chain phenotype of the neoplastic component; histopathological diagnosis was carried out according to the WHO classification; tumor stage (extent of BM infiltration by myeloma cells) was evaluated as follows: stage I: less than 20% (7 cases), stage II: 20-50% (7 cases), stage III: >50% (7 cases). Double immunohistochemistry of κ-light chain with HES6 or IL-6 was performed by a standard avidin-biotin-peroxidase complex technique on slides with consecutive serial sections cut from each BM biopsy. Antigens were visualized using alkaline phosphatase (κ-light chain in red) and diaminobenzidine (DAB) (HES6 and IL-6 in brown) as chromogens ([74] and *Supplementary Materials* for details).

### Statistical analysis

Data are represented as means ± SD from at least 3 independent experiments. Statistical analyses were performed as follows: for single culture and co-culture experiments using MM and BMSC cell lines we used two-tailed Student's t-test to compare the means of normally distributed values and analysis of variance was performed by one-way ANOVA with Tukey's post-test. For co-culture experiments on human primary cells one-way ANOVA with Bonferroni post-test was performed.

In GEP experiments Kruskal-Wallis test was applied to measure the differential expression of the selected genes between groups, Dunn's test was exploited to perform nonparametric pairwise multiple comparisons between the independent groups and Benjamini-Hochberg correction was applied to adjust significance of multiple testing [75, 76].

## SUPPLEMENTARY MATERIAL FIGURES AND TABLE


