# Supplementary material for: Multiple myeloma-derived Jagged ligands increases autocrine and paracrine interleukin-6 expression in bone marrow niche
Source: Oncotarget. 2016 Jul 24;7(35):56013–29. doi: 10.18632/oncotarget.10820 (PMC5302893; doi:10.18632/oncotarget.10820)
Supplement: Supplementary file 1 [file oncotarget-07-56013-s001.pdf]

Multiple myeloma-derived Jagged ligands increases autocrine and paracrine interleukin-6 expression in bone marrow niche

Supplementary Information

INVENTORY

Supplemental Information contains the Supplemental Data (12 figures) and Supplemental experimental Procedures.

<u>Supplemental Data</u>:

Figure S1 is related to Fig1

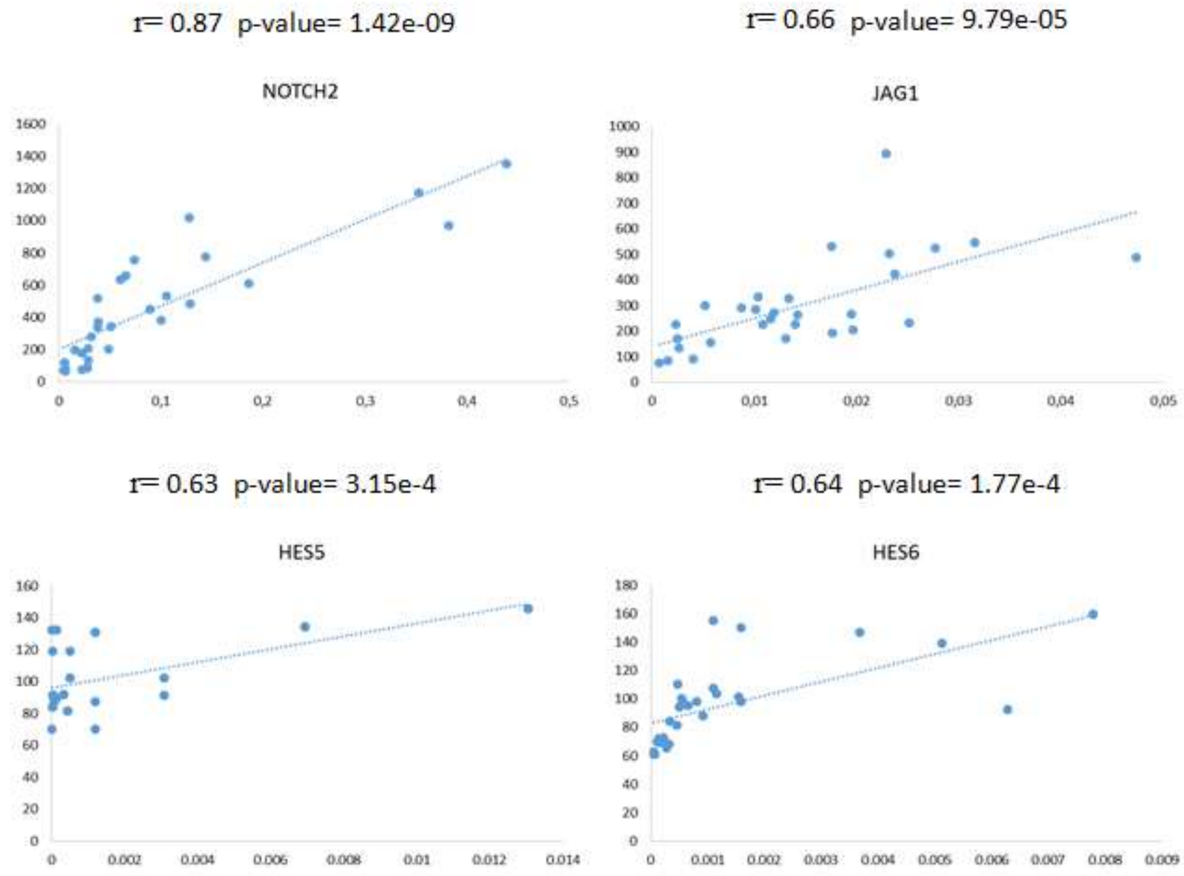

Figure S1: Quantitative RT-PCR validation of GEP data for NOTCH genes pathway in 30 MM cases. *NOTCH2*, *JAG1*, *HES5* and *HES6* expression validation; Pearson's correlation coefficient (r) was calculated between GEP data (y-axis) and quantitative RT-PCR results (x-axis) expressed as 2^{Ct}. Significant positive correlation between GEP and qRT-PCR data are evidenced by Pearson's coefficient r>0.60 with a p-value<0.05.

Figures S2, S3, S4 and S5 are related to Figure 2.

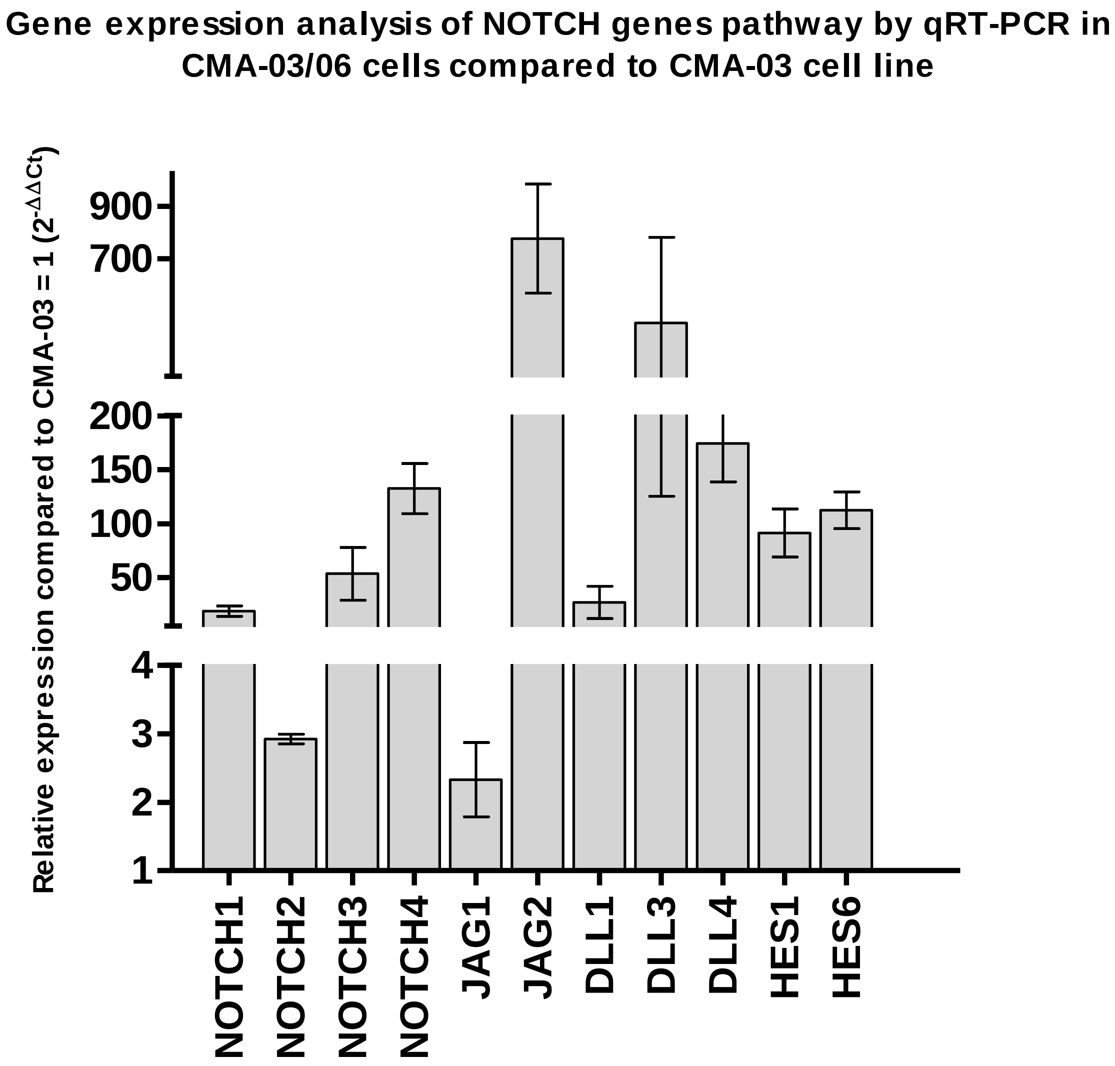

Figure S2: Notch pathway genes expression levels in CMA-03/06 cells compared to CMA-03. The relative gene expression of Notch pathway genes (normalized to GAPDH) in CMA-03/06 was assessed by qRT-PCR. Results were obtained by comparing CMA-03/06 cells to CMA-03 by the $2^{-\Delta\Delta Ct}$ formula. Graph shows the mean values ± SD.

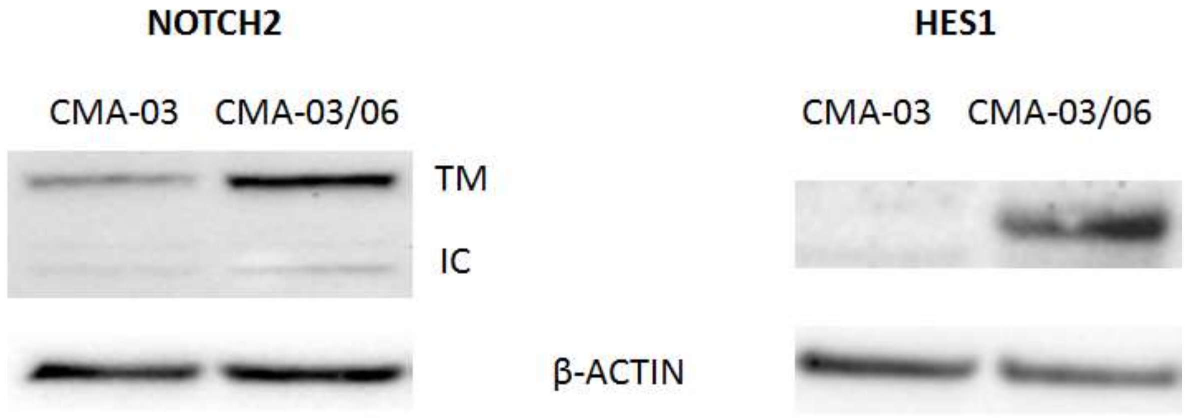

Figure S3: Western blot analysis of Notch2 and HES1 in CMA-03 and CMA-03/06 cells. Protein expression level of transmembran (TM) and active intracellular (IC) Notch2 and HES1 in CMA-03 and CMA-03/06. Notch2 and HES1 are overexpressed also at protein level in CMA-03/06 compared to CMA-03. Representetive results of three independent experiments.

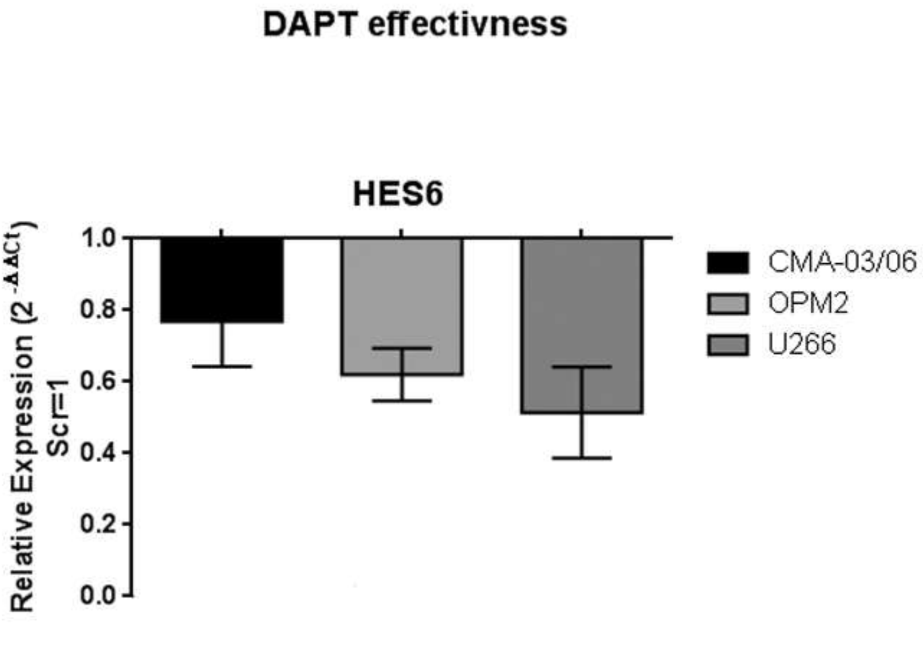

Figure S4: DAPT inhibits Notch activity in CMA-03/06, OPM2 and U266 cell lines. The relative gene expression of HES6 (normalized to GAPDH) in CMA-03/06, OPM2 and U266 cells treated with 50µM DAPT was assessed by qRT-PCR. Results were obtained by comparing DAPT-treated cells to DMSO by the $2^{-\Delta\Delta Ct}$ formula. Graph shows the mean values ± SD.

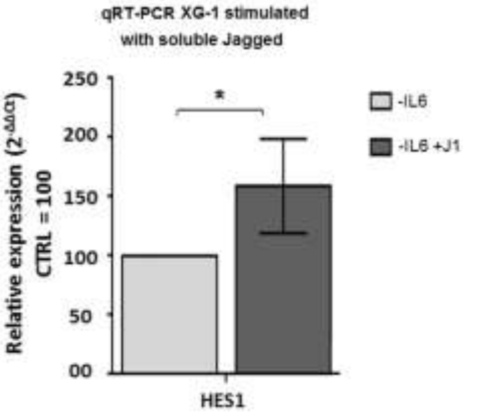

Figure S5: Jag1 synthetic peptide activates the Notch pathway in MM cells. Confirmation of JAG1 stimulation effectiveness was obtained by qPCR measure of relative HES1 gene expression variation in absence of IL-6 in JAG1 stimulated cells compared to untreated, calculated by the $2^{-\Delta\Delta Ct}$ formula. SD were calculated from 3 independent experiments. Mean value ± SD are shown. Statistical analysis by t-test: *= $p<0.05$.

Figures S6, S7 and S8 are related to Figure 3.

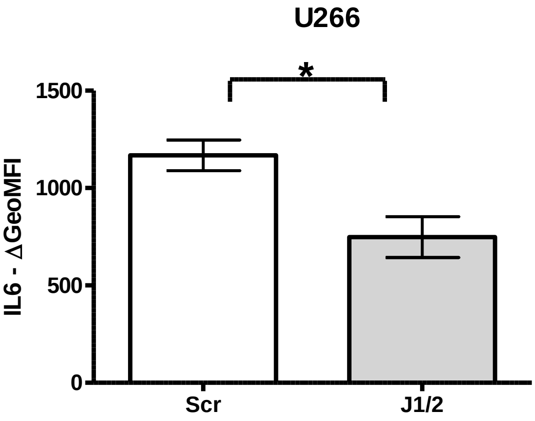

Figure S6: Jag1/2 silencing causes a decrease in IL6 production by MM cells. The graph shows the mean values of GeoMFI obtained from the flow cytometric analysis of IL6 in U266-J1/2KD (J1/2) or U266-SCR (Scr) cells. For every sample the appropriate isotype control was subtracted from the positive signal. The bars are the mean values of GeoMFI from 3 independent experiments. Statistical analysis was performed by two-tailed t-test (*=$p<0.05$).

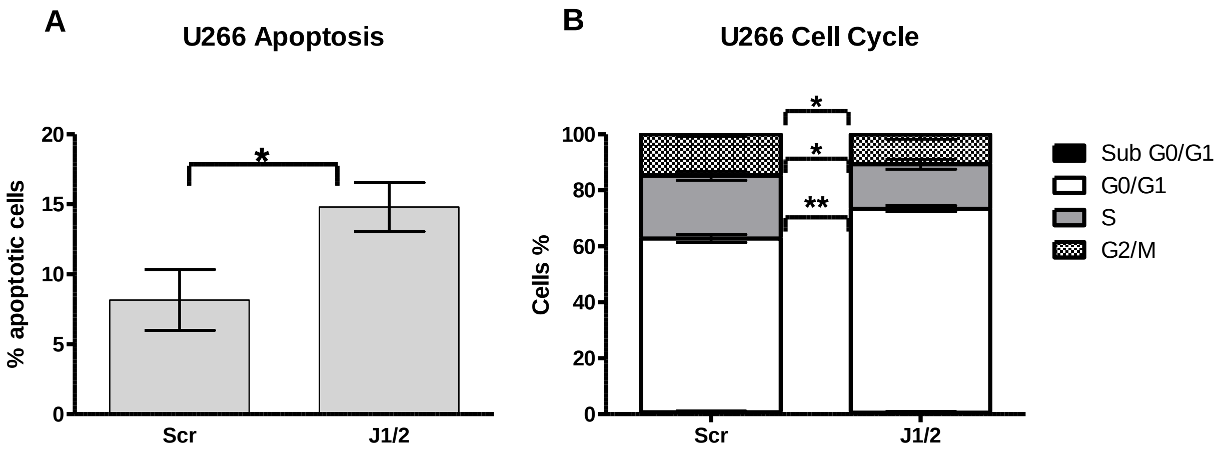

Figure S7: Effects of Jag1/2 silencing of U266 cells apoptosis and cell cycle. A) Annexin-V and PI double staining was performed to measure the apoptotic rate of MM cells treated with anti-Jag1/2 siRNAs. Statistical analysis was performed by two-tailed t-test (*=p<0.05). B) Cell cycle analysis. A standard PI staining was used to measure cell cycle distribution based on cell DNA content. Means and standard deviations were calculated on three independent experiments and statistical analysis was performed by two-tailed t-test (*=p<0.05; **=p<0.01).

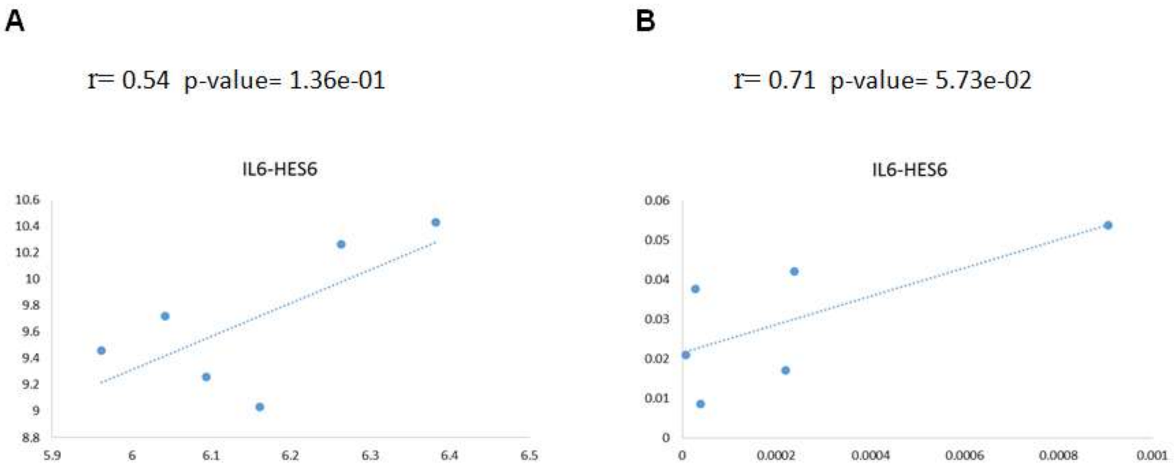

Figure S8: Correlation of *IL-6* and Notch activity in MM patients with high IL-6 expression levels. Panel A: Correlation plot of *IL-6* (y-axis) and *HES6* (x-axis) expression levels (log2 scale) measured by microarray gene expression profile in 6 MM patients of the proprietary GEO dataset No. GSE66293, showing the highest *IL-6* gene expression levels. One-tailed Pearson's correlation coefficient (r) and the corresponding p-value are reported. **Panel B:** Correlation plot of *IL-6* (y-axis) and *HES6* (x-axis) expression levels ($2^{-\Delta Ct}$ method) measured by qRT-PCR in the same 6 MM patients of panel A. One-tailed Pearson's correlation coefficient (r) and the corresponding p-value are reported.

Figures S9 and S10 are related to Figure 4.

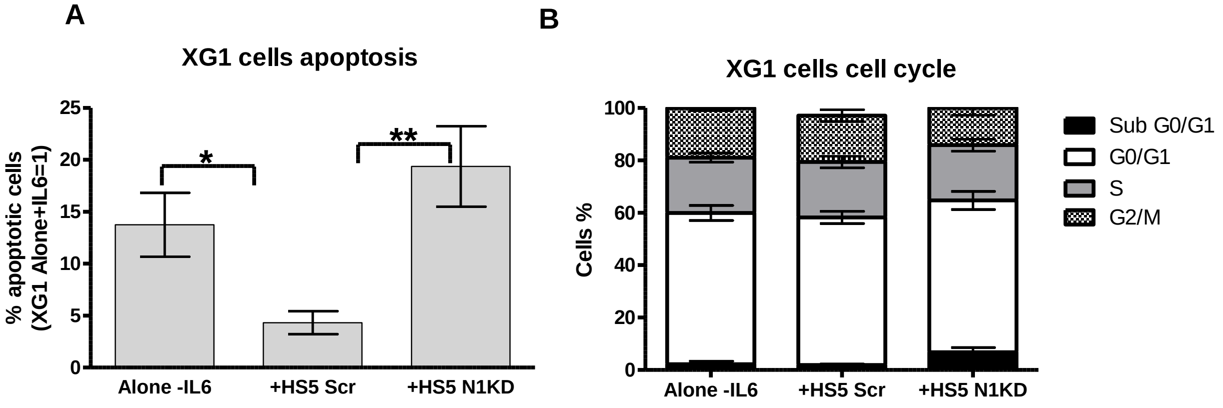

Figure S9: Effects of Notch1 silencing on HS5 cells ability to promote XG1 cells growth and survival. A) Annexin-V-APC staining was performed to measure the apoptotic rate of MM cells co-cultured with Scr or N1KD HS5. The presence of GFP in HS5 cells allowed to discriminate the two populations in flow cytometry. Results were normalized on apoptosis in XG1 cultured alone in presence of IL6. Statistical analysis was performed with one-way ANOVA (*=p<0.05; **=p<0.01). **B)** Cell cycle analysis. DRAQ5 was used to measure cell cycle distribution of GFP-negative cells based on cell DNA content. Means and standard deviations were calculated on three independent experiments. Statistical analysis, performed with one-way ANOVA, failed to detect statitically significan changes in the cell cycle of XG1 cells.

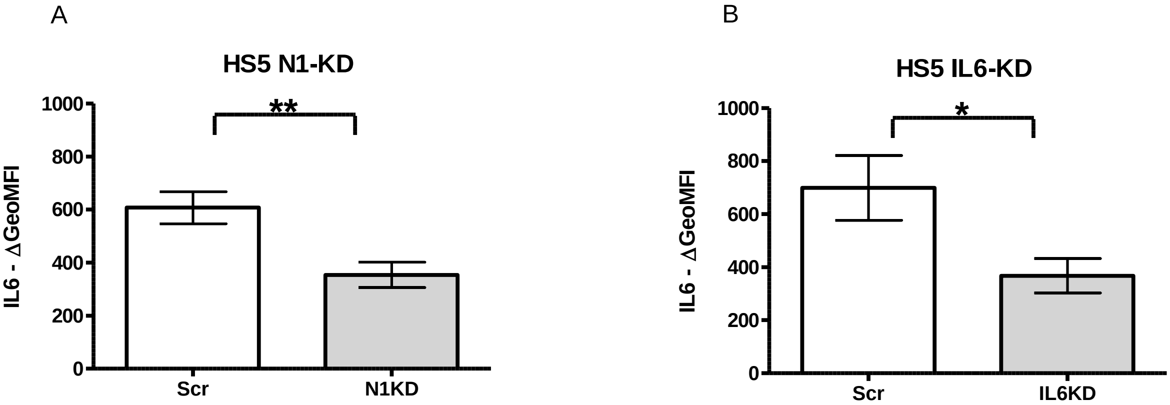

Figure S10: Effect of Notch or IL6 silencing on IL6 production by HS5 cells. The graphs show the mean values of GeoMFI obtained from the flow cytometric analyses of IL6 in HS5-N1KD cells (Panel A) or HS5-IL6KD (Panel B) compared to HS5-SCR. For every sample the appropriate isotype control was subtracted from the positive signal in order to obtain the ΔGeoMFI. The bars are the mean values of GeoMFI from 4 independent experiments for Notch1 kockdown and 5 experiments for IL-6 knockdown. Statistical analysis was performed by two-tailed t-test (*=p<0.05; **=p<0.01).

Figure S11 and S12 are related to Figure 5.

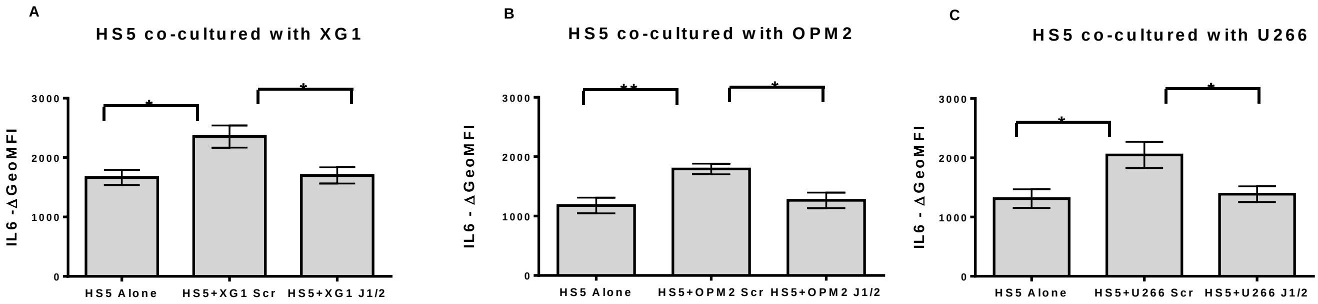

Figure S11: Jag1/2 silencing causes a decrease in MM cells ability to promote IL6 production by stromal cells. The graphs show the mean value of GeoMFI obtained from the flow cytometric analysis of IL6 in HS5 cells co-cultured with HMCLs-J1/2KD or HMCLs-SCR cells. Specifically, co-coltured HMCLs were XG1 (A), OPM2 (B) and U266 (C) cell lines. For every sample the appropriate isotype control was subtracted from the positive signal. The bars are the mean values of GeoMFI from 3 independent experiments for XG1, 3 for OPM2 and 4 for U266 cells. Statistical analysis was performed with one-way ANOVA and Bonferroni post-test (*=p<0.05; **=p<0.01).

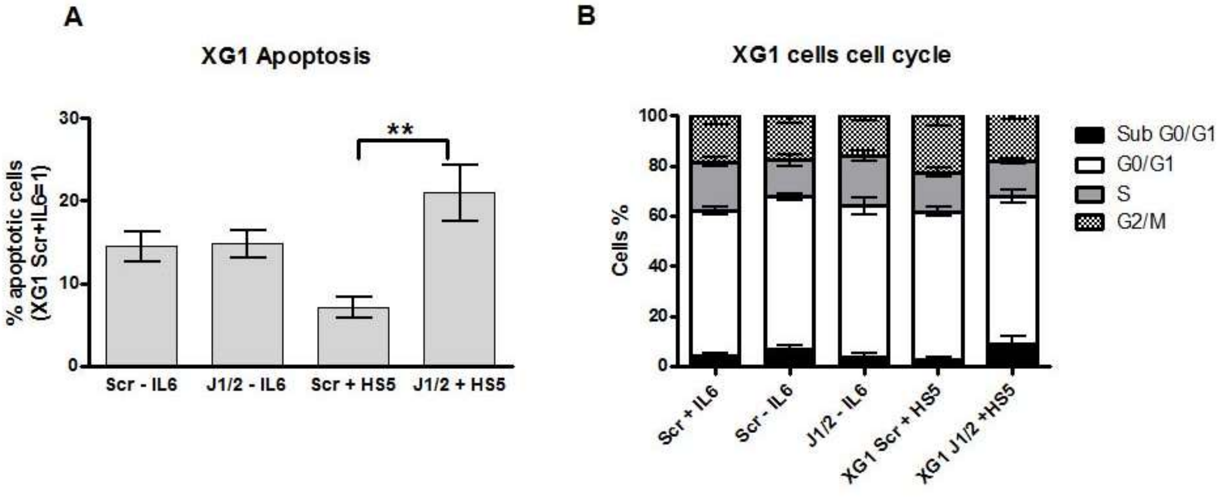

Figure S12: Effects of Jag1/2 silencing on HMCLs apoptosis and cell cycle. A) Annexin-V-APC staining was performed to measure the apoptotic rate of XG1-J1/2KD or XG1-SCR cells co-cultured with GFP+ HS5 cells. Results flow cytometric analysis were normalized on apoptosis in XG1 Scr cultured alone in presence of IL6. Statistical analysis was performed with one-way ANOVA (*=p<0.05). **B)** DRAQ5 was used to measure cell cycle distribution of GFP- cells based on cell DNA content. Means and standard deviations were calculated on three independent experiments. Statistical analysis, performed with one-way ANOVA, failed to detect statitically significan changes in the cell cycle of XG1 cells.

<u>Supplemental experimental Procedures</u>

RT-qPCR Primers

Here are reported all the primer sets used for the RT-qPCR analysis.

RT-qPCR primers	Forward Primer 5’-3’	Reverse Primer 5’-3’
**mGAPDH**	TTGGCCGTATTGGGCGCCTG	CACCCTTCAAGTGGGCCCCG
**mHES5**	GGCTCACCCCAGCCCGTAGA	TCGTGCCCACATGCACCCAC
**mIL6**	TGAACAACGATGATGCACTTGCAGA	TCTCTGAAGGACTCTGGCTTTGTCT
**hGAPDH**	ACAGTCAGCCGCATCTTCTT	AATGGAGGGGTCATTGATGG
**hNOTCH1**	GGCGGGAAGTGTGAAGCGGC	GTGGCATGTCCCGGCGTTCT
**hNOTCH2**	AGACCATTTTGCCAATCGAG	GTGCTTCAGGCTGAGGAAAG
**hNOTCH3**	GTTCATGCATTGACCTCGTG	AGCGCAAACCAGTGTATCCT
**hNOTCH4**	GAGGAAGAAGAGGGGCAGTG	ACAGGGTTCTGGGAAACTCC
**hHES1**	GATGCTCTGAAGAAAGATAGC	GTGCGCACCTCGGTATTAAC
**hHES6**	ATGAGGACGGCTGGGAGA	ACCGTCAGCTCCAGCACTT
**hHES5**	GTGGAGAAGATGCGCCGCGA	AGCGTCAGGAACTGCACGGC
**hJAG1**	TTCGCCTGGCCGAGGTCCTAT	GCCCGTGTTCTGCTTCAGCGT
**hJAG2**	CCGGCCCCGCAACGACTTTT	CCTCCCTTGCCAGCCGTAGC
**hDLL1**	GGTGGGCAGGTACAGGAGTA	TATCCGCTATCCAGGCTGTC
**hDLL3**	TCACCTCCAATCTGGTCTCC	TCCCAGAATTTCAAACCCAA
**hDLL4**	CCTGTCCACTTTCTTCTCGC	ACTACTGCACCCACCACTCC
**hIL6**	TTCAATGAGGAGACTTGCCTGGTGA	TCTGCACAGCTCTGGCTTGGTTCC

Table 1: Primer sequences.

HES6 and IL-6 immunohistochemistry

Three-μm thick tissue sections were used for immunohistochemical (IHC) studies using a standard avidin-biotin-peroxidase complex technique. Briefly, sections were dewaxed, rehydrated in xylene-alcohol; antigen retrieval was performed with buffered citrate (pH 6) at 97C° for 35 min and endogenous peroxidase activity was blocked with 3% hydrogen peroxide for 10 min. Serial sections were incubated with the primary antibodies against the following targets: HES6 (polyclonal Abcam; 1: 300), IL6 (monoclonal mouse Ab, Santacruz; 1:300). Monoclonal neoplastic component was

visualized by double IHC, with κ light chain (polyclonal rabbit, DAKO Cytomation; 1: 20000). HES6 and IL6 reaction was detected in brown with the autostainer (480 Bioptical instruments Thermoscinetific) by Novolink Max polymer detection system (Leica Microsystem, Italy), after incubation with diaminobenzidine (Novolink-NOVOCASTRA); light chain was visualized in red (Ultravision Quanto Detection System AP polymer, Thermoscientific); sections were then counterstained with hematoxylin. Negative control slides were processed without primary antibody. IHC examination of the slides was performed independently by two pathologists; in case of discordant grouping attribution, slides were re-evaluated and discussed till inter-observer concordance. Images were acquired with the NanoZoomer-XR C12000 series (HAMAMATSU PHOTONICS K.K.).

Detection of apoptosis

Cells were washed with cold PBS1x, resuspended in "Binding buffer 1X" (HEPES 0,01M, NaCl 0,14M, CaCl2 2,5mM) and incubated for 15' with Annexin-V FITC (Immunotools, DE) + Propidium Iodide (2,5 ug/ml final, Sigma-Aldrich) in the dark. Finally, 400 µl of Binding Buffer 1x were added to the tube and samples were processed and analyzed using the BD FACSVerse™ System (BD Biosciences).

Cell cycle analysis

Cells were washed with cold PBS and resuspended in "GM Buffer" 1x (glucose 1mg/ml, EDTA 0,2 mg/ml, 2% FBS in PBS). Cells were fixed by adding Ethanol (70% final) and incubated O.N. at 4°C. Then, samples were washed in PBS 5% FBS and incubated in Staining Buffer (RNAse 25µg/ml (Applichem), Propidium Iodide (Sigma Aldrich) 25µg/ml, NP-40 (Sigma Aldrich) 0,004% in PBS) O.N. at 4°C. Cells were processed and analyzed using the BD FACSVerse™ System (BD Biosciences).

Analysis of cell cycle and apoptosis in co-culture systems

To allow to discriminate between MM cells and BMSCs in flow cytometry, before co-culture and treatments HS5 were stained with PKH26 (Sigma Aldrich).

Apoptosis analysis were performed as reported above using Annexin-V APC(Immunotools).

Cell cycle analysis was performed using DRAQ5 staining (ThermoFisher Scientific) according to accordingly to manufacturer instructions. Cells were processed and analyzed using the BD FACSVerse™ System (BD Biosciences).
